# Metatranscriptomic-driven insights into mucosal glycan degradation by the human gut microbiota

**DOI:** 10.1093/femsec/fiaf118

**Published:** 2025-12-04

**Authors:** Franziska Bauchinger, David Berry

**Affiliations:** Division of Microbial Ecology, Department of Microbiology and Ecosystem Science, Centre for Microbiology and Environmental Systems Science, University of Vienna, 1030 Vienna, Austria; Doctoral School in Microbiology and Environmental Science, University of Vienna, 1030 Vienna, Austria; Division of Microbial Ecology, Department of Microbiology and Ecosystem Science, Centre for Microbiology and Environmental Systems Science, University of Vienna, 1030 Vienna, Austria; Joint Microbiome Facility of the Medical University of Vienna and the University of Vienna, 1030, Vienna, Austria

**Keywords:** *Akkermansia muciniphila*, *Bacteroides thetaiotaomicron*, *Bacteroides vulgatus*, cooccurrence network analysis, linear regression analysis, response–predictor networks

## Abstract

The secreted mucus layer in the human gastrointestinal tract constitutes both a protective boundary between gut lumen and epithelium as well as an important nutrient source for members of the gut microbiota. While many gut microbes possess the genetic potential to degrade mucin, it is still unclear which species transcribe the respective genes. Here, we systematically analysed publicly available metagenome and metatranscriptome datasets to characterize the gut microbial community involved in mucosal glycan degradation. We utilized cooccurrence network analysis and linear regression to elucidate the ecological strategies of, and relationship between, mucus degraders. We found that although ~60% of species carrying genes encoding for mucosal-glycan-degrading enzymes have detectable transcription of these genes, only 21 species prevalently transcribe more than 1 gene. Furthermore, the transcription of individual genes was frequently dominated by single species in individual samples. Transcription patterns suggested the presence of competitive mucosal glycan degraders characterized by abundance-driven transcription that were negative predictors for the transcription of other degraders as well as opportunistic species with decoupled abundance and transcription profiles. These findings provide insights into the ecology of the mucosal glycan degradation niche in the human gut microbiota.

## Teaser

Metatranscriptomics and modeling provide insights into the ecology of the mucosal glycan degradation niche by the the human gut microbiota.

## Introduction

The human gastrointestinal (GI) tract is inhabited by a diverse community of microorganisms that form the human gut microbiota. These microbes provide essential functions to their human host, such as metabolizing dietary compounds (Hamaker and Tuncil 2014), conferring resistance to pathogens (Brugiroux et al. 2016), and immunomodulation (Zheng et al. 2020). The microbiota reside in the gut lumen and are prevented from invading the gut epithelium in part via the action of the secreted mucus layer. Mucin, the main component of secreted mucus, is a heavily glycosylated glycoprotein that is secreted by goblet cells in the epithelium of the GI tract. An inner, denser mucus layer is virtually free of microbes while a looser outer layer functions as a habitat for members of the gut microbiota (Johansson et al. 2008). Some gut microbes can also utilize the glycans that form the mucosal structure as an energy source (Paone and Cani 2020). The mucus layer of the GI tract is formed from branched, cross-linked glycan chains consisting of galactose (Gal), *N*-acetylgalactosamine (GalNAc), *N*-acetylglucosamine (GlcNAc), fucose (Fuc), and sialic acid (Neu5Ac) (Podolsky 1985, Robbe et al. 2004). These glycan chains, which represent around 80% of mucin mass (Gendler and Spicer 1995), are largely linked through GalNAc moieties to serine or threonine residues of the mucus protein backbone (Brockhausen et al. 2009). While the exact structure and length of the glycan chains can vary considerably, the terminal sugars are either GalNAc, Fuc, or Neu5Ac (Podolsky 1985, Brockhausen et al. 2009, Tailford et al. 2015). Gal or GlcNAc are additionally frequently sulphated (Luis et al. 2021).

Degradation of these complex structures requires a multitude of different carbohydrate-active enzymes (CAZymes). Bioinformatic analysis of the genomic potential of gut microbiota to degrade mucosal glycans has predicted that up to 86% of this microbial community harbor genes linked to mucus degradation (Ravcheev and Thiele 2017). Few species, however, encode a larger array of the necessary enzymes and are able to extensively degrade mucin, such as *Akkermansia muciniphila* (Shuoker et al. 2023) or *Bacteroides thetaiotaomicron* (Kostopoulos et al. 2021). As has been shown experimentally, mucus degradation is usually a communal process and individual species are capable of breaking only specific linkages between glycans (Pudlo et al. 2015, Raimondi et al. 2021). Individual glycan-derived sugars can also be available to nondegraders as many mucosal glycan degradation enzymes act extracellularly (Yamaguchi and Yamamoto 2023). *Akkermansia muciniphila*, for example, produces a number of sialidases and fucosidases, enabling it to remove terminal sugars and thereby not only facilitating further degradation of glycans, but also sharing nutrients with cooccurring microbes (Shuoker et al. 2023). *Ruminococcus gnavus* has been shown to liberate mucosal glycans and make them accessible to *B. thetaiotaomicron* (Schaus et al. 2024), a species viewed as a glycan generalist that encodes multiple mucus degradation CAZymes but shows a preference for other substrates (Pudlo et al. 2015).

An imbalance between mucus production by the epithelial cells and degradation by the microbiome has been linked to various health issues such as colitis (Fang et al. 2021) and colorectal cancer (Coleman and Haller 2021), and thinning of the mucus layer may be linked to dietary fiber deprivation (Jegatheesan et al. 2024). Understanding both the composition of the microbial community that degrades mucosal glycans as well as the ecological principles governing the mucus degradation guild is consequently of high interest. The complex mucosal structure makes it a nutrient source that may support an equally complex microbial community and allow for the coexistence of both highly specialized and competitive mucus degraders as well as opportunistic microbes. Mucus degradation is an interplay between the production of glycans by the gut epithelium, competition of gut microbes for these glycans (Kostopoulos et al. 2021), and the necessity for collaborative degradation due to individual microbes’ inability to degrade the complete mucosal structure (Arike and Hansson 2016, Shuoker et al. 2023). As such it can provide interesting insights into the ecological properties of gut microbial communities.

Previous work made it apparent that a surprisingly large number of gut microbes are theoretically able to degrade mucosal structures, while work on individual mucin degraders and cocultures furthered our understanding of the underlying ecological strategies. However, we lack knowledge on the properties of mucus degrading communities. It is as of yet unclear, which community members transcribe mucosal glycan degradation enzymes in the GI tract and how these species navigate the apparent overlap in ecological niche. Here, we aimed to address some of these open questions through the analysis of publicly available metatranscriptomes from stool samples of healthy adults. We investigated which mucin degradation enzymes can be detected in the transcriptomes and identified microbial species that prevalently transcribe these enzymes. We then analysed whether abundance and transcriptional patterns of mucosal glycan degraders are linked to elucidate differences in ecological strategy.

## Materials and methods

### Processing of raw data

We analysed the paired metagenome and metatranscriptome reads from 574 stool samples of two publicly available datasets: a cohort of male health professionals (Mehta et al. 2018), and a study on inflammatory bowel disease (Schirmer et al. 2018), focusing on the healthy control participants in the second dataset. We processed the raw reads with trimmomatic (Bolger et al. 2014) (leading: 3, trailing: 3, slidingwindow: 4:15, minlen: 50), removed samples with under 1 million reads and analysed the trimmed reads with HUMAnN 3.9 (Beghini et al. 2021). Within HUMAnN 3.9, we here used MetaphlAn 3.1 (Beghini et al. 2021) to estimate community composition, map reads to a community pangenome with bowtie2 (version 2.5) (Langmead and Salzberg 2012) and align unmapped reads to a protein database using DIAMOND 2.1.9 (Buchfink et al. 2015). This combination allows for an analysis of both the taxonomic and the functional profiles of metagenome and metatranscriptome reads. We used the MetaphlAn setting rel_ab_w_read_stats to obtain both relative abundances as well as estimated read counts in the taxonomic profiles. In the functional profiles, reads were mapped to gene families and we further grouped them into enzyme commission numbers (ECs) with the HUMAnN function humann_regroup_table and the flag—-groups uniref90_level4ec. Gene family and EC values are given as copies per million.

### Identification of ECs involved in mucin glycan degradation

We investigated mucosal glycan degradation on the level of ECs, a classification scheme for enzymes based on the catalysed reaction. We identified ECs of interest through a literature review by cross-referencing glycosyl hydrolase families presumed to be involved in mucin glycan degradation, as reviewed by Ravcheev and Thiele (2017), Berkhout et al. (2022) as well as Glover et al. (2022), with the corresponding entries in the Carbohydrate Active Enzymes (CAZy) database (https://www.cazy.org/) (Drula et al. 2022) and in InterPro (Paysan-Lafosse et al. 2023). In addition to glycosyl hydrolases (GH), we also looked at sulfatases involved in mucin degradation (Berkhout et al. 2022). The corresponding ECs of the sulfatases were obtained from the Kyoto Encyclopedia of Genes and Genomes (Kanehisa and Goto 2000). The distribution of reads grouping to the respective ECs was visualized utilizing k-means clustering with the function kmeans in the R package stats (Dalgaard 2010). In order to compare a species’ mucosal glycan degradation gene repertoire with their overall capacity to hydrolyze glycans, we extracted all ECs corresponding to GH families (EC 3.2.1.-) (Drula et al. 2022) from the metagenomes and metatranscriptomes. The investigated ECs and corresponding GH families can be found in Table S1.

### Identification of mucosal glycan degraders

We compiled all species encoding any of the identified ECs and then went on to filter for species transcribing these ECs. We removed species only transcribing Alpha- and/or Beta-galactosidase from further analysis due to the broad dissemination of galactose. We also did not further consider species only transcribing one of the identified ECs and removed species transcribing ECs in <10% of samples with detectable transcription. We chose this cut-off based on a sensitivity analysis performed for correlation networks that has shown that a sample size of 20 samples or more is needed for robust network inference (Berry and Widder 2014). These steps resulted in a list of versatile (transcribing more than 1 EC) as well as prevalent (transcribing at least 1 EC in at least 10% of samples with detectable transcription) mucosal glycan degraders. The distribution of both the encoding and transcribing species amongst phylogenetic groups was visualized in a radial cladogram. The cladogram was built with the function ggtree from the R package of the same name (Xu et al. 2022) and species were grouped based on order, family, and genus.

### Cooccurrence network of mucus degraders

We computed species cooccurrence networks to better understand abundance patterns of mucus degraders. These networks are based on Spearman correlations between the centered log-ratio (clr) transformed estimated read counts of species. We used centered log-ratio transformation to account for the compositional nature of metagenome sequencing data (Kucera and Malmgren 1998). For each sample, we divided the estimated read counts by the geometric mean and then log-transformed the ratio, adding a pseudocount of 1 to avoid nonfinite numbers. Then we computed pairwise Spearman correlations between species with these log-transformed ratios using the R function cor() with method = “spearman.” We estimated the significance of the observed correlations by comparing them to a null distribution. This null distribution was obtained by randomly shuffling read counts and calculating Spearman correlations from the shuffled data 1000 times. We considered observed correlations to be significant if they were outside the 99% confidence interval of the null distribution. We then removed all negative correlations as well as positive correlations <0.2 and filtered for species correlated to any of the investigated mucosal glycan degraders (as defined above). We chose these conservative cut-offs to ensure statistically significant and biologically meaningful correlations. This resulted in a network of species exhibiting a significant and strong cooccurrence pattern with the mucosal glycan degraders. We used the function cluster_fast_greedy from the R package igraph (Csárdi et al. 2023) on the adjacency matrix of the network (based on presence/absence of correlations) to further cluster these species according to their cooccurrence patterns. The cooccurrence network was visualized with Cytoscape 3.10.1 (Shannon et al. 2003), with network nodes representing species and edges (connections between nodes) representing significant positive correlations between mucosal glycan degraders and their first neighbors (species with a positive correlation to a mucosal glycan degrader). A node and edge table of the computed cooccurrence network is available in Table S2.

### Linear models between species abundance and transcription

In order to understand the relationship between the abundance of mucosal glycan degraders and their contribution to transcription of degradation enzymes we utilized linear models. For each investigated EC, we computed linear models between the abundance of each prevalently contributing species (clr transformed estimated read numbers) and their respective contribution to EC transcription (% of total EC transcription per sample). Linear model statistics are reported in Table S3. We considered a model with a *P*-value < .05 as significant and used the adjusted *R*² values to estimate how much variation in transcription is explained by the abundance patterns of the respective species.

### Response–predictor networks of EC transcription

We further utilized linear models to understand the relationship between mucosal glycan degraders. Specifically, we computed linear models for each investigated EC to estimate how the individual abundance patterns of prevalently transcribing species (detectable transcription in at least 10% of samples where a respective EC is transcribed) in turn influence the transcription patterns of each of those transcribing species. For each EC the linear model looks as follows: *Transcription species 1 [% of total EC transcription] ∼ abundance species 2 [clr transformed read counts] + … + abundance species n [clr transformed read counts]*. Significant coefficients in the linear model indicate that transcription of the response species (species 1) is predicted by the abundance of a predictor species (species 2–n). The detailed linear model statistics can be found in Table S4. We again used Cytoscape 3.10.1 (Shannon et al. 2003) to visualize these linear model coefficients as edges in response–predictor networks. Lastly, we built a consensus network combining all edges across response–predictor networks of investigated mucosal-glycan-degrading enzymes.

## Results

### Ten ECs involved in the degradation of mucosal glycans are prevalent in the analysed metagenomes and metatranscriptomes

**Figure 1.**
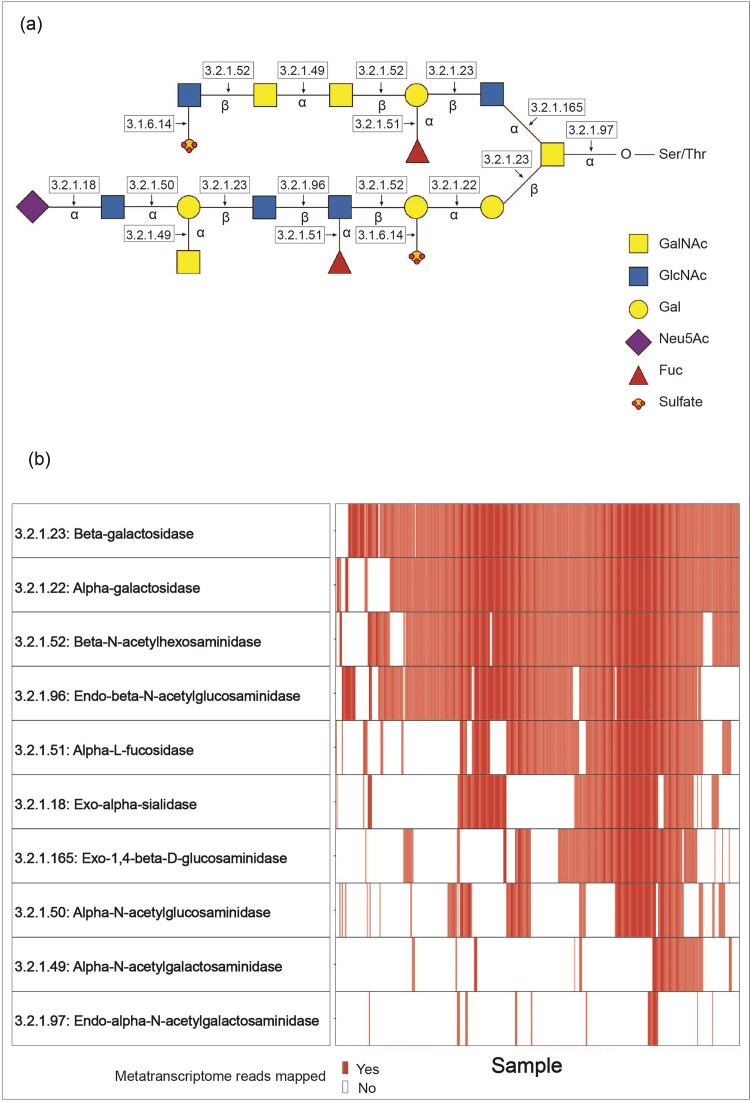
Enzymes involved in the degradation of mucosal glycans. (A) Hypothetical mucosal glycan structure and respective degradative enzymes (indicated with the corresponding enzyme commission numbers = ECs). (B) Distribution of ECs across analysed metatranscriptomes. Samples with reads grouping to a respective EC are shown in red. Samples are ordered based on a k-means clustering algorithm.

In previous work, a large suite of enzymes has been identified as being potentially involved in the degradation of mucosal glycans (Ravcheev and Thiele 2017, Berkhout et al. 2022, Glover et al. 2022). In this study, we analysed 574 paired metagenomes and metatranscriptomes (Mehta et al. 2018, Schirmer et al. 2018) at the level of ECs to evaluate whether the genes and transcripts encoding these enzymes could be detected in the stool microbiomes of healthy adults. We screened for 18 ECs, comprising 13 glycosyl hydrolases, 1 lyase, and 4 sulfatases (Table S1) and analysed their abundance and prevalence. In only 10 of the analysed metatranscriptome samples, none of the ECs were detected. From the total of 18 ECs, 11 were detectable in the analysed dataset (Table 1) and 10 ECs were prevalently (prevalence >5%) detected (Fig. 1). Reads aligning to EC 3.1.6.14 were only found in ∼1% of samples and could not be mapped to any reference genomes. We therefore did not include this enzyme in further analyses.

**Table 1.** Prevalence and mean abundance of reads mapped to ECs of mucosal glycan degradation enzymes in metagenomes and metatranscriptomes and number of species encoding and transcribing these ECs.

	Metagenomes			Metatranscriptomes		
EC	%	cpm	#	%	cpm	#
3.1.6.14: *N*-acetylglucosamine-6-sulfatase	1	0.05	0	1	0.05	0
3.2.1.165: Exo-1,4-beta-d-glucosaminidase	100	51.42	35	42	1.34	20
3.2.1.18: Exo-alpha-sialidase	99	19.65	19	43	1.90	13
3.2.1.22: Alpha-galactosidase	100	398.25	268	87	16.70	164
3.2.1.23: Beta-galactosidase	100	799.49	322	94	45.70	201
3.2.1.49: Alpha-*N*-acetylgalactosaminidase	94	11.20	11	16	0.26	10
3.2.1.50: Alpha-*N*-acetylglucosaminidase	94	39.56	9	34	0.78	9
3.2.1.51: Alpha-l-fucosidase	98	21.87	28	57	3.05	17
3.2.1.52: Beta-*N*-acetylhexosaminidase	100	187.31	177	85	17.90	85
3.2.1.96: Endo-beta-*N*-acetylglucosaminidase	99	32.12	36	80	16.98	21
3.2.1.97: Endo-alpha-*N*-acetylgalactosaminidase	30	0.35	6	5	0.11	4

EC: Enzyme commission number.

%: Prevalence (%).

cpm: Abundance (copies per million).

#: Number of species mapped.

### A small subset of bacteria encoding mucosal-glycan-degrading enzymes transcribe these genes

**Figure 2.**
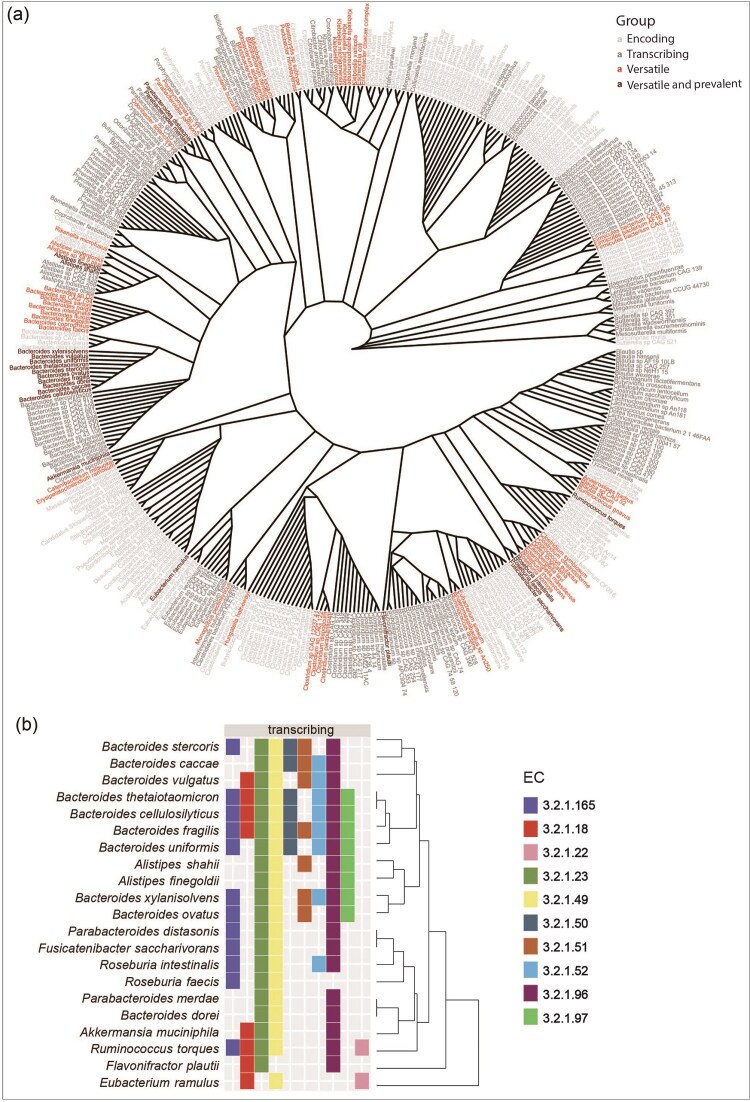
Species encoding and transcribing mucosal glycan degradation enzymes. (A) Radial cladogram of species encoding the analysed ECs (light gray), transcribing any of the ECs (dark gray), transcribing more than 1 EC (light red), and prevalently transcribing (transcription prevalence ≥10%) more than one EC (dark red). (B) Species prevalently transcribing more than one EC. Species are ordered based on their EC transcription profile. The dendrogram indicates which species have the most similar EC profiles.

We identified 395 species, belonging to 49 different families, that encoded one or more of the detected ECs. However, only 238 of these species, belonging to 33 different families, transcribed at least 1 gene in the analysed dataset (Fig. 2A). An overview of which species encode and transcribe which ECs is given in Fig. S1. We excluded species transcribing only one of the compiled ECs to focus on a subset of species that we consider versatile mucin degraders. Additionally, we excluded species only transcribing alpha-galactosidase and/or beta-galactosidase. Both enzymes cleave off galactose and considering that galactose is a sugar widely occurring in dietary compounds and the focus of this study is the degradation of mucosal glycans, we chose not to further consider these species. Lastly, we only focused on species transcribing at least 1 EC in at least 10% of the analysed samples. This resulted in 21 versatile as well as prevalent mucosal glycan degraders (Fig. 2B), belonging to the families *Akkermansiaceae* (1 species), *Bacteroidaceae* (10 species), *Eubacteriaceae* (1 species), *Lachnospiraceae* (4 species), *Rikenellaceae* (2 species), *Ruminococcaceae* (1 species), and *Tannerellaceae* (2 species) (Fig. 2B). The average contribution of each degrader to the transcription of the analysed ECs is shown in Fig. S2.

### Mucosal glycan degraders form distinct clusters with cooccurring species

**Figure 3.**
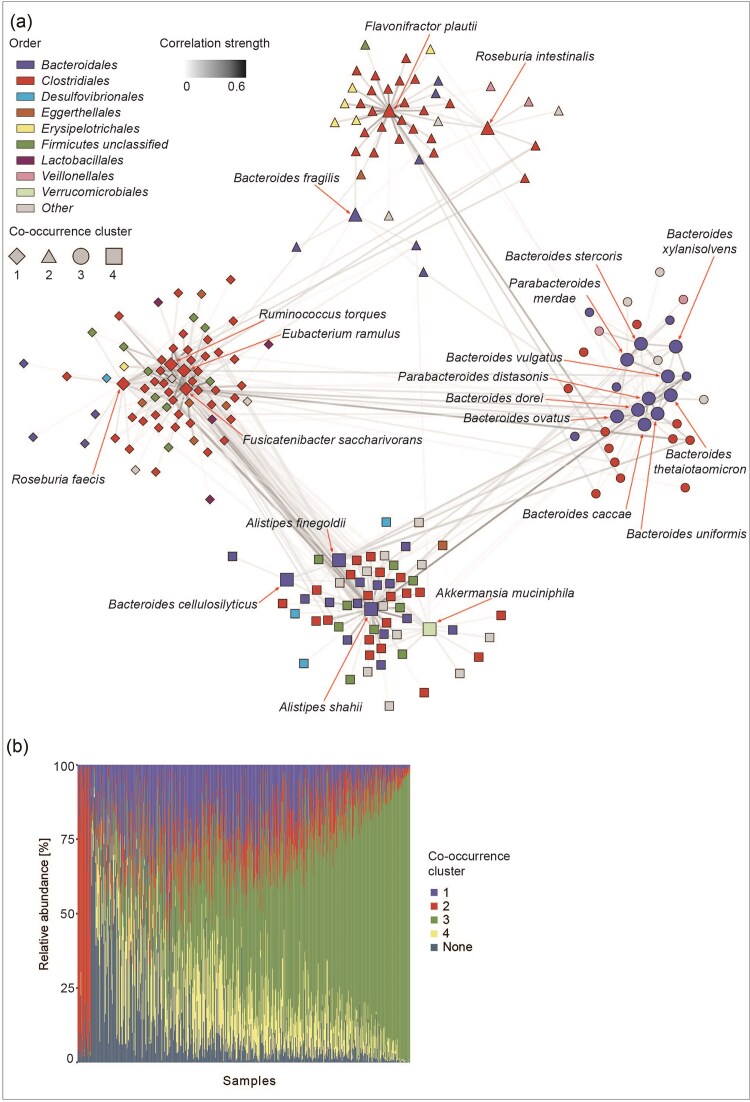
Cooccurrence clusters of mucosal glycan degraders and their first neighbors. (A) Cooccurrence network of mucosal glycan degraders and their first neighbors (species positively correlated with a mucosal glycan degrader). Network nodes depict species and network edges are significant positive correlations. Node shape indicates the cooccurrence cluster, node color indicates phylogenetic order of a species, and edge color indicates correlation strength. Nodes of mucosal glycan degraders are larger and labeled. (B) Relative abundance (%) of cooccurrence network clusters across samples. Colors indicate the respective cluster that species were grouped into. Samples were ordered based on a k-means clustering algorithm.

We next investigated with which species mucosal glycan degraders cooccur. We computed a cooccurrence network based on Spearman correlations and performed a clustering analysis to identify the species with which glycan degraders consistently cooccur. This revealed four distinct clusters (Fig. 3A). Cluster 1 is the largest cluster and contains four mucosal glycan degraders, namely *Roseburia faecis, Fusicatenibacter saccharivorans, Ruminococcus torques*, and *Eubacterium ramulus*, all of which belong to the order *Clostridiales*. In total, 44 out of 71 species in this cluster are *Clostridiales*. Cluster 2 contains 3 degraders, *Flavonifractor plautii, Bacteroides fragilis*, and *Roseburia intestinalis*. Again, most species, 25 out of 44, belong to the order *Clostridiales*. Cluster 3 is the smallest cluster but contains the largest number of degraders (10 species), all of which belong to the order *Bacteroidales*. The mucosal glycan degraders in this cluster are *Bacteroides uniformis, Parabacteroides distasonis, Bacteroides xylanisolvens, Bacteroides ovatus, Bacteroides caccae, Bacteroides vulgatus, Parabacteroides merdae, Bacteroides stercoris, B. thetaiotaomicron*, and *Bacteroides dorei*. Out of 33 species in this cluster, 15 belong to the order *Bacteroidales* and 12 to the order *Clostridiales*. The last cluster, cluster 4, contains four mucin degraders, *Bacteroides cellulosilyticus, Alistipes finegoldii, Alistipes shahii*, and *A. muciniphila. Clostridia* and *Bacteroidales* are again the dominant orders in this cluster of 61 species, with 20 and 16 species, respectively. We observed the highest number of between-cluster positive correlations between clusters 2 and 4 while cluster 1 has the lowest number of positive correlations to any other cluster (Fig. 3A). None of the clusters are mutually exclusive, and in fact they frequently all cooccur in the analysed samples (Fig. 3B). However, we observed a gradient from samples heavily dominated by a single cluster to samples with a very evenly distributed composition.

### Transcription of individual genes is often dominated by a single species

**Figure 4.**
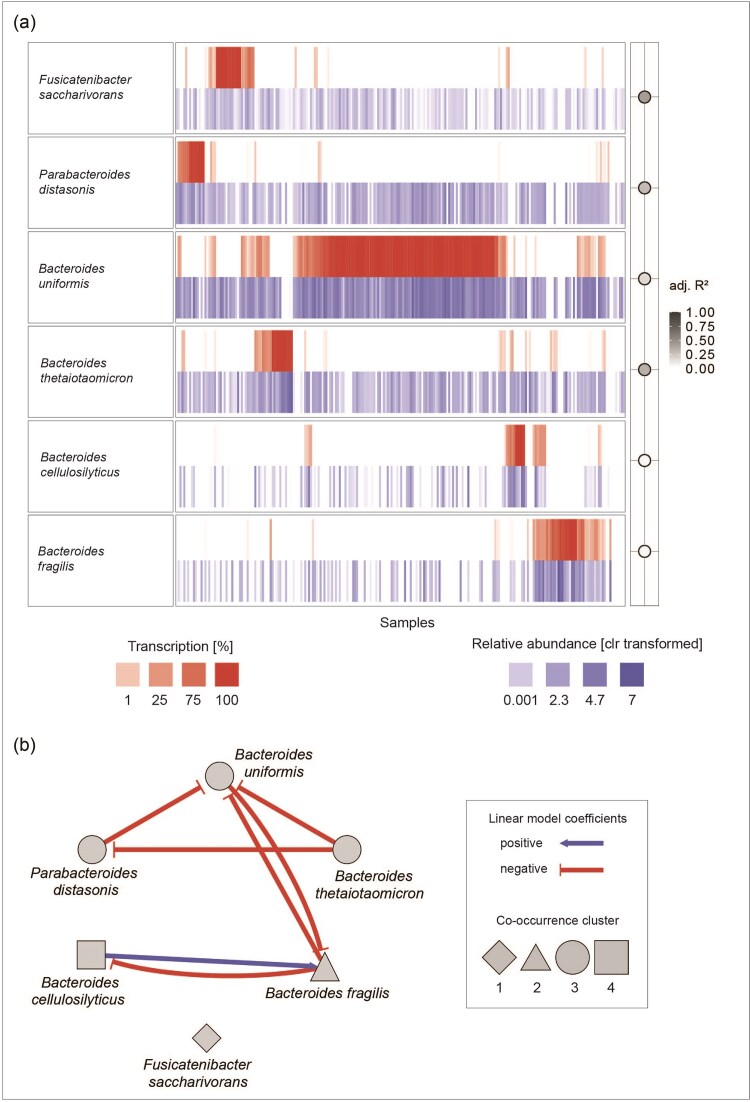
Contribution of species to transcription of EC 3.2.1.165: Exo-1,4-beta-d-glucosaminidase. (A) Transcription of EC 3.2.1.165 mapped to individual species (in % of total transcription grouped to EC 3.2.1.165) is shown in red. Relative abundance (clr transformed) of prevalently transcribing (≥10%) species is shown in blue. Percentage of abundance-driven transcription (= adjusted *R*² of linear models between species abundance and transcription) is shown in gray circles on the right. (B) Response–predictor network of EC 3.2.1.165 transcription. Nodes are prevalently transcribing species and node shape indicates the cooccurrence cluster a species belongs to (Fig. 3A). Edges are significant coefficients of linear models (*Transcription response species [% of total EC transcription] ∼ abundance predictor species 1 [clr transformed read counts] + … + abundance predictor species n [clr transformed read counts]*). Edges are directed from predictor to response species. Inhibition (negative coefficient) is indicated in red and facilitation (positive coefficient) is indicated in blue.

In order to investigate what percentage of EC transcription is attributed to individual species, we normalized the transcription levels per species to total transcription attributed to the respective EC per sample. This analysis revealed an interesting common pattern in all investigated ECs: transcription is frequently entirely dominated by a single species, but the dominant species can vary between samples (Fig. 4A and Figs S3A–S11A). We then evaluated if this pattern is simply reflecting the abundance of the respective species. If this were the case, species abundance should be a strong predictor for transcription levels. Linear models between species abundance and species transcription of individual ECs (in percentage of total transcription attributed to the respective EC) resulted in explained proportions of variance (adjusted *R*²) ranging from −0.02 to 0.53, with an overall median of 0.16 (Table S3). In general, species abundance explains the highest proportions of transcriptional variance (median >0.2) in *B. dorei, A. muciniphila, B. stercoris, B. uniformis, A. finegoldii, R. intestinalis, B. cellulosilyticus*, and *B. fragilis* (Table S3). We refer to species with a high proportion of abundance-driven transcription as competitive degraders and species with a low proportion of abundance-driven transcription as opportunistic degraders. While abundance is a good predictor for the contribution to transcription in competitive species, it can only partially explain the observed transcription patterns.

### The abundance patterns of other degraders partially explain transcription patterns

**Figure 5.**
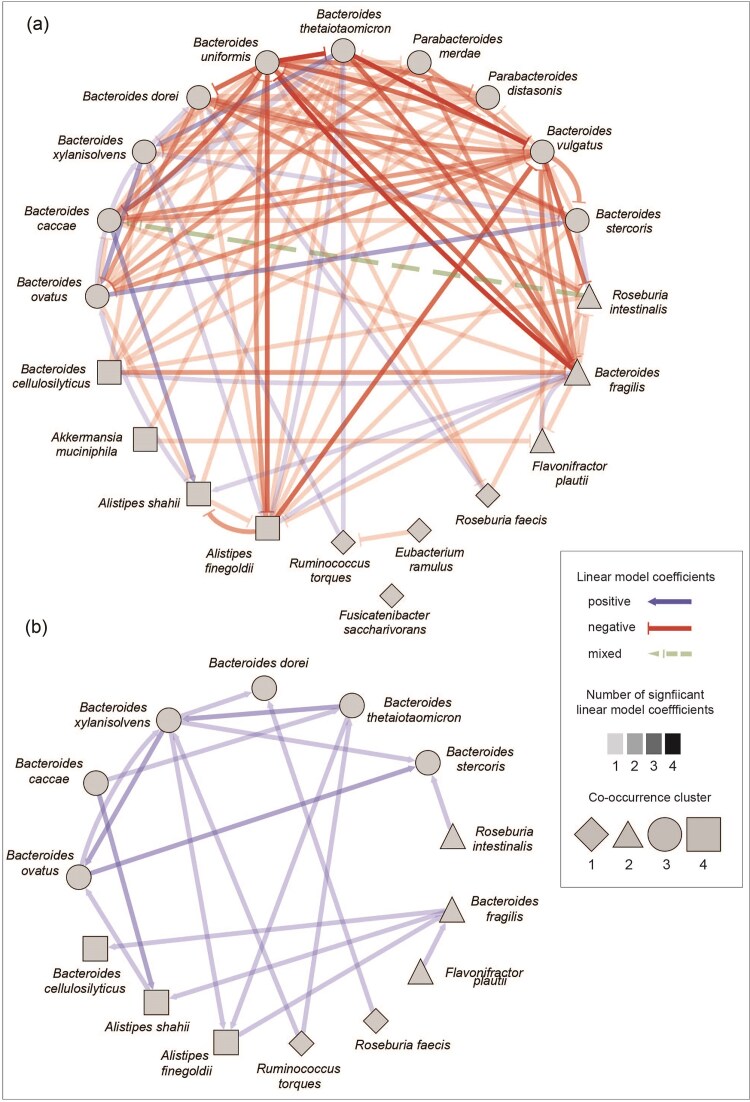
Consensus response–predictor networks of all analysed ECs. Nodes are mucosal glycan degraders and node shape indicates the cooccurrence cluster a species belongs to (Fig. 3A). Edges are directed from predictor to response and edge color and shape indicates whether the association between species across networks is consistent (blue—always positive, red—always negative) or mixed (green). Edge transparency depicts the number of significant linear model coefficients across response–predictor networks (light gray—low, dark gray—high). (A) Consensus network summarizing positive and negative associations between species. (B) Consensus network depicting only positive associations between species.

We next evaluated if the abundance of other species transcribing the same enzyme can further explain or predict the transcription of a given species. To evaluate this, we computed linear models of the form: *transcription species 1 [% of total EC transcription] ∼ abundance species 2 [clr transformed abundance] + abundance species 3 [clr transformed abundance] + … + abundance species n [clr transformed abundance]*. If the abundance of another transcribing species (species 2–n = predictor species) can explain some of the variance in the transcription level of the species in question (species 1 = response species) this species will be a significant predictor in the linear model with the coefficient indicating the effect size. A positive coefficient indicates that the transcription of the response species will be high if the abundance of the predictor species is high. A negative coefficient indicates that the transcription of the response species is low if the abundance of the predictor species is high. In order to visualize all significant predictors in these linear models, we built response–predictor networks for each EC (Fig. 4B and Figs S3B–S11B). Statistical details on these linear models can be found in Table S4. In general, we predominantly observed negative associations between species, where the abundance of one species is a negative predictor for the transcription of another species. Positive relationships between species occur particularly in alpha- and beta-galactosidase as well as alpha-*N*-acetylglucosaminidase, perhaps indicating a higher potential for facilitating interactions in more widely disseminated glycans. In order to summarize all response–predictor networks of individual ECs we built a consensus network (Fig. 5A). This consensus network visualizes the number of times a significant relationship between two species was observed in the individual networks and whether they are consistently positive, negative or mixed. Interestingly, there was only one case of a mixed relationship across all 10 individual networks: the abundance of *R. intestinalis* is a negative predictor for *B. caccae*’s transcription of alpha-l-fucosidase and a positive predictor for its transcription of alpha-galactosidase. This may indicate generally stable relationships between mucosal glycan degraders. In four individual networks there was a negative relationship from *B. uniformis* to *B. thetaiotaomicron*, from *B. thetaiotaomicron* to *B. vulgatus* and from *B. fragilis* to *B. uniformis*, suggesting strong competition between these degraders. Positive relationships between species are observed much less frequently, occurring in a maximum of two individual networks (Fig. 5B). We observe reoccurring positive relationships from *B. xylanisolvens* to *B. ovatus*, from *B. thetaiotaomicron* to *B. xylanisolvens*, from *B. ovatus* to *B. stercoris*, and from *B. caccae* to *A. shahii*. This may indicate either direct or indirect enhancement of transcription by the presence of another species. In summary, we see very stable relationships between mucosal glycan degraders when investigating the transcription of multiple enzymes.

### Competitive mucosal glycan degraders are associated with reduced transcriptional activity of other degraders

**Figure 6.**
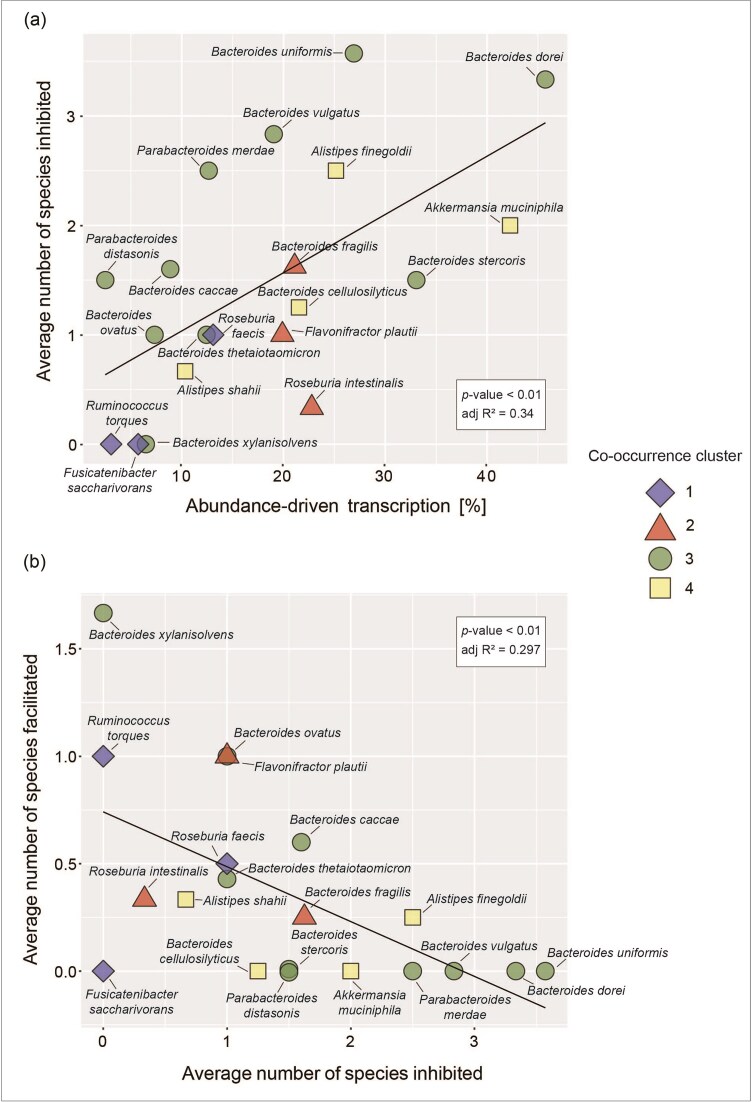
Abundance-driven transcription and transcription inhibition and facilitation in prevalent mucosal glycan degrading species. Shape and color of data points refers to the cooccurrence cluster the species belong to (Fig. 3A). (A) Percentage of abundance-driven transcription (= median *R*² of linear models between species abundance and transcription) versus average number of species inhibited (= average number of significant negative linear model coefficients in response–predictor networks) (Linear model: *P*-value < .01, adjusted *R*² = 0.34). (B) Average number of species inhibited (= average number of significant negative linear model coefficients in response–predictor networks) versus average number of species facilitated (= average number of significant positive linear model coefficients in response–predictor networks) (Linear model: *P*-value < .01, adjusted *R*² = 0.297).

To further elucidate how abundance and transcription patterns of mucosal glycan degraders are connected, we compared the predictive power of a species' own abundance on its transcription patterns with this species effect on other species transcription (Fig. 6A). This revealed that more competitive degraders (high percentage of abundance-driven transcription) tend to be associated with reduced transcriptional activity of a larger number of species. Conversely, the abundance of less competitive degraders is rarely a negative predictor for the transcription of other species. Additionally, we found that negative and positive associations between species are inversely correlated (Fig. 6B), meaning that the less competitive a degrader is the more likely it is to be associated with increased transcription of other degraders. Finally, we evaluated whether competitive mucosal glycan degraders have distinctive genomic features associated with polysaccharide or glycan degradation. We found a consistent correlation between the number of mucosal glycan degradation enzymes and the number of other glycosyl hydrolase families that degraders encode and transcribe (Fig. S12A and B, respectively). This indicates that all investigated species seem to have a similar proportion of mucosal glycan degradation enzymes in comparison to their repertoire of glycosyl hydrolases.

## Discussion

Many members of the human gut microbiome encode enzymes that are predicted to have mucosal glycan degradation activities (Ravcheev and Thiele 2017, Glover et al. 2022). However, we observe that a much smaller portion is transcribing these genes in a given community and even fewer species transcribe them prevalently across the analysed samples (Fig. 2B). Frequently the transcription of a particular gene is attributed to a single species in individual samples (Fig. 4A, Figs S3–S11A). In a proportion of prevalent mucosal glycan degraders, this seems linked to their abundance patterns. For other species their own abundance is not a good predictor of transcription, but their transcription patterns are frequently linked to the absence or low abundance of other species (Fig. 5A). We speculate that this reflects a continuum between competitive and opportunistic degraders. Competitive degraders tend to show a high percentage of abundance-driven transcription (Fig. 6A), meaning transcription of mucosal glycan degradation genes is high when a species is abundant. Opportunistic degraders, on the other hand, do not transcribe these genes whenever they are abundant, but seem to be only sporadically involved in mucosal glycan degradation. This decouples their abundance and transcription patterns, resulting in a low percentage of abundance-driven transcription (Fig. 6A).

Additionally, the presence of competitive degraders tends to be associated with inhibited transcription of other degraders (Fig. 6A) while opportunistic degraders tend to facilitate it (Fig. 6B). While negative associations seem to be predominant in the mucosal glycan degrading community (Fig. 5A), we do find indications for facilitation. Notably, this is the case for *R. torques*. The abundance of *R. torques* is a positive predictor for the transcription of beta-galactosidase in both *B. thetaiotaomicron* and *B. xylanisolvens* (Fig. S6B). It has previously been shown that *R. torques* can liberate products from larger mucin structure, making them accessible for *B. thetaiotaomicron* (Schaus et al. 2024). Our results suggest that not only may other species, such as *B. xylanisolvens*, also benefit from this behavior, relationships like these may be more widespread. In particular, we find that the abundance patterns of *B. xylanisolvens, B. caccae, B. ovatus*, and *B. fragilis* are positive predictors for transcription patterns of multiple other species, indicating that they may facilitate mucus degradation in other members of the community. Another well-known mucin degrader that shows a strong prevalence for mucosal glycans is *A. muciniphila*. It can express beta-galactosidase and beta-hexosaminidase (Kostopoulos et al. 2021) as well as sialidases and fucosidases (Shuoker et al. 2023). *Akkermansia muciniphila* has also been shown to upregulate defense-related genes in the presence of other mucin degraders (Kostopoulos et al. 2021), suggesting that it is a competitive mucus degrader. Although we only found *A. muciniphila* to prevalently transcribe exo-alpha-sialidase in the analysed metatranscriptomes, we still observe a strong link between the species’ abundance and its transcription of exo-alpha-sialidase (Table S3). Additionally, the abundance of *A. muciniphila* is a negative predictor for the transcription of this enzyme by both *B. thetaiotaomicron* and *F. plautii* (Fig. S3B), perhaps reflecting *A. muciniphila*’s competitive advantage in mucus degradation. We observe similar patterns in *B. fragilis*, a species that exhibits a preference for O-glycans over starch and inulin (Pudlo et al. 2015), although *B. fragilis* seems to be less competitive than *A. muciniphila* as it shows a lower percentage of abundance-driven transcription and on average inhibits fewer species (Fig. 6A). *Bacteroides thetaiotaomicron*, on the other hand, was previously described as a mucin degradation generalist (Kostopoulos et al. 2021) and is able to rapidly respond to multiple glycans (Rogers et al. 2013). Correspondingly, we find that *B. thetaiotaomicron* prevalently transcribes 7 out of the 10 investigated mucin degradation enzymes, underlining its generalist strategy. It has also been shown that *B. thetaiotaomicron* downregulates polysaccharide utilization loci associated with mucin degradation in the presence of other polysaccharides (Kostopoulos et al. 2021), suggesting an opportunistic rather than competitive role in mucin degradation. The abundance of *B. thetaiotaomicron* is, in contrast to *B. fragilis* and *A. muciniphila*, a poor predictor for its transcription patterns (Fig. 6A and Table S3), again congruent with its more opportunist role. *Bacteroides thetaiotaomicron* may have to resort to active mucin degradation when its preferred nutrient sources are scarce and competition for mucosal glycans consequently higher.

The observation that the abundance of strong competitors for mucosal glycans, as determined by their high percentage of abundance-driven transcription, tends to frequently be a negative predictor for the transcription patterns of other species (Fig. 6A) might indicate that these competitive degraders are more specialized for mucin glycan degradation and may additionally have an inhibiting effect on species degrading the same glycans. Conversely, negative associations might also indicate that opportunistic degraders take advantage of mucus products and monosaccharides liberated by the activity of competitive degraders and are therefore not transcribing any of their own mucus degradation enzymes. Such cross-feeding on mucosal glycans has previously been shown between the mucus degraders *R. torques* and *B. thetaiotaomicron* (Schaus et al. 2024) and has also been observed between mucus degraders and nondegraders, such as *Bifidobacterium bifidum* and *Bifidobacterium breve* (Egan et al. 2014) and *A. muciniphila* and *Roseburia inulinivorans* (Pichler et al. 2020). de Ram et al. (2025) further showed that porcine gastric mucin glycans could partially be degraded by mono- and cocultures of *A. muciniphila, R. torques*, and *B. thetaiotaomicron *. However, complete degradation could only be achieved by a larger mucin-degrading synthetic community. Opportunistic degraders may however be forced to produce their own enzymes to cleave off mucosal glycans in the absence, or low abundance, of more competitive degraders. We observe a particularly high number of such negative associations between mucin degraders of the genus *Bacteroides* (Fig. 5A), suggesting either strong competition for mucin glycans, common cross-feeding or even cooperative degradation. The majority of these closely related species are in the same cooccurrence cluster, cluster 3 (Fig. 3), meaning that they are frequently observed in the same samples. Tuncil et al. (2017) could show that *B. ovatus* and *B. thetaiotaomicron* exhibit different priorities for various dietary glycans, promoting stable coexistence despite their similar ecological niches. We find that the species in cooccurrence cluster 3 are spread out along the observed continuum between competitive and opportunistic mucosal glycan degraders (Fig. 6A), pointing toward differing ecological strategies that allow for cooccurrence. The previous work on nutrient prioritization (Rogers et al. 2013, Schwalm III et al. 2017, Tuncil et al. 2017) as well as work on diauxic growth of microbes (Wang et al. 2021) indicates that at least some members of the human gut microbiome strongly favor particular glycans and that this prioritization is preserved in communities as well. Interestingly, the species investigated in these studies show characteristics of mucin degradation opportunists in our analysis. We observe recurring associations of the same nature between species across transcription patterns of several enzymes. These recurring associations are mostly negative and particularly driven by species showing characteristics of competitive mucosal glycan degraders. In a complex community, the effect of nutrient prioritization in opportunistic degraders may be less apparent due to the presence of more competitive community members. However, it has to be noted that in a complex gut microbial community the glycosyl hydrolases investigated are not exclusively used to degrade mucosal glycans. In particular, GlcNAc and Gal are widely disseminated sugars that can also be found in dietary glycans (Ravcheev and Thiele 2017). Consequently, there is an overlap between the mucus and dietary polysaccharide degradation niches and communities and associations between mucus degraders could therefore also be a product of a shift between these niches. We identified a higher number of species to prevalently transcribe the GlcNAc- and Gal-cleaving enzymes as opposed to other enzymes, aligning with the likely higher prevalence of these sugars in the gut. We also primarily observe the positive associations in the response–predictor networks of these enzymes. Positive associations could therefore be the result of a shift to dietary polysaccharides when other mucin degraders are more abundant, comparable to *B. thetaiotaomicron*’s ability to switch between multiple glycans (Rogers et al. 2013). Interestingly, we find a strong linear relationship between mucosal glycan degradation genes and other glycosyl hydrolase degradation genes across all analysed species, both in terms of encoded as well as transcribed genes (Fig. S12). Some competitive degraders, such as *B. vulgatus* or *B. dorei* encode and transcribe a large number of glycosyl hydrolase degradation genes, suggesting a generalist rather than specialist strategy. Other competitive degraders, such as *A. muciniphila* and *A. finegoldii*, seem to have a much smaller number of glycosyl hydrolase degradation genes and a comparatively large number of mucosal glycan degradation genes, suggesting a specialization toward mucosal glycan degradation.

In this work we identify, in line with previous results, various members of the genus *Bacteroides* as well as other species such as *A. muciniphila* and *R. torques* as prevalent mucosal glycan degraders (Ravcheev and Thiele 2017, Berkhout et al. 2022). In particular, *B. fragilis, B. thetaiotaomicron*, and *B. cellulosilyticus* contribute to transcription of many mucin degradation enzymes (Fig. 2C). Other known mucin degraders, such as *R. gnavus* and *Bifidobacterium* spp. (Crost et al. 2016, Bell et al. 2019, Glover et al. 2022), were found to transcribe various mucin degradation genes, but at very low prevalence. Furthermore, we did not observe transcription of fucosidase in *A. muciniphila*, despite experimental evidence to the contrary (Shuoker et al. 2023). These inconsistencies may be attributed to strain variability within species or low annotation quality of certain genes and are a clear limitation of mapping metatranscriptome reads to reference genomes. Future experimental work should be done to confirm the biochemical activities of enzymes encoded by genes investigated in this study. We would also like to emphasize that the analysed publicly available datasets are from two cohorts with limited geographical diversity and therefore do not accurately represent the global diversity found in human gut microbiomes. The data analysed does also not include samples from infants, where *Bifidobacterium* spp. would likely play a much larger role in the mucus degrading community (Yamaguchi and Yamamoto 2023). Samples from the distal colon also do not reflect the entirety of the GI tract and neither can they provide insight into spatial variability (Duncan et al. 2021, Mondragón-Palomino et al. 2022). However, work on a 15-member consortium of human gut bacterial strains in gnotobiotic mice revealed that the colonic community is mixed at the micrometer scale and can be compared to an incompletely mixed bioreactor (Mark Welch et al. 2017). Mark Welch et al. (2017) further found that mucosa-attached microbes are frequently shed alongside the mucus layer and expelled with the lumen content, suggesting that stool samples, while not a perfect representation of gut content, reflect the microbial community of both the lumen and mucus layer. Lastly, the analyses presented here rely on bioinformatic analysis and experimental validation is ultimately needed to elucidate predicted interspecies relationships and ecological mechanisms.

## Conclusion

In this study, we systematically interrogated metagenome and metatranscriptome datasets for the presence of reads associated with mucosal glycan degradation enzymes. A large group of species encodes such genes, but only few species with limited diversity, mainly *Bacteroidales* and *Clostridiales*, prevalently transcribe them. We could show that transcription of the individual enzymes is frequently dominated by a single species in a given sample. In a subgroup of degraders, transcription is mostly driven by abundance suggesting that they are competitive mucosal glycan degraders while the transcription of opportunistic degraders is decoupled from their abundance. Competitive degraders are additionally frequent negative predictors for transcription of other degraders while opportunistic degraders show indications for facilitating transcription of other species. These observations further our understanding of the ecological strategies of mucosal glycan degraders and suggest a continuum between competitive and opportunistic degraders that allows cooccurrence despite a seemingly strongly overlapping ecological niche.

## Supplementary Material

fiaf118_Supplemental_Files

## Data Availability

Publicly available datasets were analysed in this study. This data is deposited in the Sequence Read Archive under BioProject ID PRJNA354235 (https://www.ncbi.nlm.nih.gov/bioproject/PRJNA354235/) and BioProject ID PRJNA389280 (https://www.ncbi.nlm.nih.gov/bioproject/PRJNA389280/).
