# Supplementary material for: Metatranscriptomic-driven insights into mucosal glycan degradation by the human gut microbiota
Source: FEMS Microbiol Ecol. 2025 Dec 4;102(1):fiaf118. doi: 10.1093/femsec/fiaf118 (PMC12721379; doi:10.1093/femsec/fiaf118)
Supplement: fiaf118_Supplemental_Files [file fiaf118_supplemental_files.zip › Supplementary_files_legends.docx]

**Supplementary figure 1.** Species encoding (left column) and transcribing (right column) mucosal glycan degradation enzymes. Species are ordered based on their EC profiles in the metagenomes and metatranscriptomes. The dendrogram indicates which species have the most similar EC profiles.

**Supplementary figure 2.** Average contribution of species to transcription of mucosal glycan degradation enzymes across analyzed samples [%]. Contribution of species transcribing an enzyme in < 10% of samples is summarized in the category “Other”.

**Supplementary figure 3.** Contribution of species to transcription of EC 3.2.1.18: Exo-alpha-sialidase.​​ (**A**) Transcription of EC 3.2.1.18 mapped to individual species (in % of total transcription grouped to EC 3.2.1.18) is shown in red. Relative abundance (clr transformed) of prevalently (>= 10%) transcribing species is shown in blue. Percentage of abundance-driven transcription (= adjusted R² of linear models between species abundance and transcription) is shown in gray circles on the right. (**B**) Response-predictor network of EC 3.2.1.18 transcription. Nodes are prevalently transcribing species and node shape indicates the co-occurrence cluster a species belongs to (Figure 3A). Edges are significant coefficients of linear models (*Transcription response species [% of total EC transcription] ~ abundance predictor species 1 [clr transformed read counts] + … + abundance predictor species n [clr transformed read counts]*). Edges are directed from predictor to response species. Inhibition (negative coefficient) is indicated in red and facilitation (positive coefficient) is indicated in blue.

**Supplementary figure 4.** Contribution of species to transcription of EC 3.2.1.22: Alpha-galactosidase​. (**A**) Transcription of EC 3.2.1.22 mapped to individual species (in % of total transcription grouped to EC 3.2.1.22) is shown in red. Relative abundance (clr transformed) of prevalently (>= 10%) transcribing species is shown in blue. Percentage of abundance-driven transcription (= adjusted R² of linear models between species abundance and transcription) is shown in gray circles on the right. (**B**) Response-predictor network of EC 3.2.1.22 transcription. Nodes are prevalently transcribing species and node shape indicates the co-occurrence cluster a species belongs to (Figure 3A). Edges are significant coefficients of linear models (*Transcription response species [% of total EC transcription] ~ abundance predictor species 1 [clr transformed read counts] + … + abundance predictor species n [clr transformed read counts]*). Edges are directed from predictor to response species. Inhibition (negative coefficient) is indicated in red and facilitation (positive coefficient) is indicated in blue.

**Supplementary figure 5.** Contribution of species to transcription of EC 3.2.1.23: Beta-galactosidase.​ (**A**) Transcription of EC 3.2.1.23 mapped to individual species (in % of total transcription grouped to EC 3.2.1.23) is shown in red. Relative abundance (clr transformed) of prevalently (>= 10%) transcribing species is shown in blue. Percentage of abundance-driven transcription (= adjusted R² of linear models between species abundance and transcription) is shown in gray circles on the right. (**B**) Response-predictor network of EC 3.2.1.23 transcription. Nodes are prevalently transcribing species and node shape indicates the co-occurrence cluster a species belongs to (Figure 3A). Edges are significant coefficients of linear models (*Transcription response species [% of total EC transcription] ~ abundance predictor species 1 [clr transformed read counts] + … + abundance predictor species n [clr transformed read counts]*). Edges are directed from predictor to response species. Inhibition (negative coefficient) is indicated in red and facilitation (positive coefficient) is indicated in blue.

**Supplementary figure 6.** Contribution of species to transcription of EC 3.2.1.49: Alpha-N-acetylgalactosaminidase.​ (**A**) Transcription of EC 3.2.1.49 mapped to individual species (in % of total transcription grouped to EC 3.2.1.49) is shown in red. Relative abundance (clr transformed) of prevalently (>= 10%) transcribing species is shown in blue. Percentage of abundance-driven transcription (= adjusted R² of linear models between species abundance and transcription) is shown in gray circles on the right. (**B**) Response-predictor network of EC 3.2.1.49 transcription. Nodes are prevalently transcribing species and node shape indicates the co-occurrence cluster a species belongs to (Figure 3A). Edges are significant coefficients of linear models (*Transcription response species [% of total EC transcription] ~ abundance predictor species 1 [clr transformed read counts] + … + abundance predictor species n [clr transformed read counts]*). Edges are directed from predictor to response species. Inhibition (negative coefficient) is indicated in red and facilitation (positive coefficient) is indicated in blue.

**Supplementary figure 7.** Contribution of species to transcription of EC 3.2.1.50: Alpha-N-acetylglucosaminidase​​. (**A**) Transcription of EC 3.2.1.50 mapped to individual species (in % of total transcription grouped to EC 3.2.1.50) is shown in red. Relative abundance (clr transformed) of prevalently (>= 10%) transcribing species is shown in blue. Percentage of abundance-driven transcription (= adjusted R² of linear models between species abundance and transcription) is shown in gray circles on the right. (**B**) Response-predictor network of EC 3.2.1.50 transcription. Nodes are prevalently transcribing species and node shape indicates the co-occurrence cluster a species belongs to (Figure 3A). Edges are significant coefficients of linear models (*Transcription response species [% of total EC transcription] ~ abundance predictor species 1 [clr transformed read counts] + … + abundance predictor species n [clr transformed read counts]*). Edges are directed from predictor to response species. Inhibition (negative coefficient) is indicated in red and facilitation (positive coefficient) is indicated in blue.

**Supplementary figure 8.** Contribution of species to transcription of EC 3.2.1.51: Alpha-L-fucosidase.​​​ (**A**) Transcription of EC 3.2.1.51 mapped to individual species (in % of total transcription grouped to EC 3.2.1.51) is shown in red. Relative abundance (clr transformed) of prevalently (>= 10%) transcribing species is shown in blue. Percentage of abundance-driven transcription (= adjusted R² of linear models between species abundance and transcription) is shown in gray circles on the right. (**B**) Response-predictor network of EC 3.2.1.51 transcription. Nodes are prevalently transcribing species and node shape indicates the co-occurrence cluster a species belongs to (Figure 3A). Edges are significant coefficients of linear models (*Transcription response species [% of total EC transcription] ~ abundance predictor species 1 [clr transformed read counts] + … + abundance predictor species n [clr transformed read counts]*). Edges are directed from predictor to response species. Inhibition (negative coefficient) is indicated in red and facilitation (positive coefficient) is indicated in blue.

**Supplementary figure 9.** Contribution of species to transcription of EC 3.2.1.52: Beta-N-acetylhexosaminidase.​​​​ (**A**) Transcription of EC 3.2.1.52 mapped to individual species (in % of total transcription grouped to EC 3.2.1.52) is shown in red. Relative abundance (clr transformed) of prevalently (>= 10%) transcribing species is shown in blue. Percentage of abundance-driven transcription (= adjusted R² of linear models between species abundance and transcription) is shown in gray circles on the right. (**B**) Response-predictor network of EC 3.2.1.52 transcription. Nodes are prevalently transcribing species and node shape indicates the co-occurrence cluster a species belongs to (Figure 3A). Edges are significant coefficients of linear models (*Transcription response species [% of total EC transcription] ~ abundance predictor species 1 [clr transformed read counts] + … + abundance predictor species n [clr transformed read counts]*). Edges are directed from predictor to response species. Inhibition (negative coefficient) is indicated in red and facilitation (positive coefficient) is indicated in blue.

**Supplementary figure 10.** Contribution of species to transcription of EC 3.2.1.96: Endo-beta-N-Acetylglucosaminidase.​ (**A**) Transcription of EC 3.2.1.96 mapped to individual species (in % of total transcription grouped to EC 3.2.1.96) is shown in red. Relative abundance (clr transformed) of prevalently (>= 10%) transcribing species is shown in blue. Percentage of abundance-driven transcription (= adjusted R² of linear models between species abundance and transcription) is shown in gray circles on the right. (**B**) Response-predictor network of EC 3.2.1.96 transcription. Nodes are prevalently transcribing species and node shape indicates the co-occurrence cluster a species belongs to (Figure 3A). Edges are significant coefficients of linear models (*Transcription response species [% of total EC transcription] ~ abundance predictor species 1 [clr transformed read counts] + … + abundance predictor species n [clr transformed read counts]*). Edges are directed from predictor to response species. Inhibition (negative coefficient) is indicated in red and facilitation (positive coefficient) is indicated in blue.

**Supplementary figure 11.** Contribution of species to transcription of EC 3.2.1.97: Endo-alpha-N-acetylgalactosaminidase.​ (**A**) Transcription of EC 3.2.1.97 mapped to individual species (in % of total transcription grouped to EC 3.2.1.97) is shown in red. Relative abundance (clr transformed) of prevalently (>= 10%) transcribing species is shown in blue. Percentage of abundance-driven transcription (= adjusted R² of linear models between species abundance and transcription) is shown in gray circles on the right. (**B**) Response-predictor network of EC 3.2.1.97 transcription. Nodes are prevalently transcribing species and node shape indicates the co-occurrence cluster a species belongs to (Figure 3A). Edges are significant coefficients of linear models (*Transcription response species [% of total EC transcription] ~ abundance predictor species 1 [clr transformed read counts] + … + abundance predictor species n [clr transformed read counts]*). Edges are directed from predictor to response species. Inhibition (negative coefficient) is indicated in red and facilitation (positive coefficient) is indicated in blue.

**Supplementary figure 12.** Glycosyl hydrolase suite and mucosal glycan degradation enzyme suite of species. (**A**) Number of GH families encoded versus number of mucosal glycan degradation enzymes encoded (Linear model: p-value

< 0.001, adjusted R² = 0.66) (**B**) Average number of GH families transcribed versus average number of mucosal glycan degradation enzymes transcribed (Linear model: p-value < 0.001, adjusted R² = 0.89).

**Supplementary table S1.** Enzyme commission numbers, names, corresponding GH families and description of catalyzed reactions of mucosal glycan degradation enzymes.

**Supplementary table S2.** Node and edge table of co-occurrence network between mucosal glycan degraders and their first neighbors.

**Supplementary table S3.** P-values, adjusted R² values and median adjusted R² values of significant (p-value < 0.05) abundance-transcription linear models.

**Supplementary table S4.** Significant (p-value < 0.05) linear model coefficients of response-predictor networks.
