# Supplementary figures and images for: Metatranscriptomic-driven insights into mucosal glycan degradation by the human gut microbiota
Source: FEMS Microbiol Ecol. 2025 Dec 4;102(1):fiaf118. doi: 10.1093/femsec/fiaf118 (PMC12721379; doi:10.1093/femsec/fiaf118)

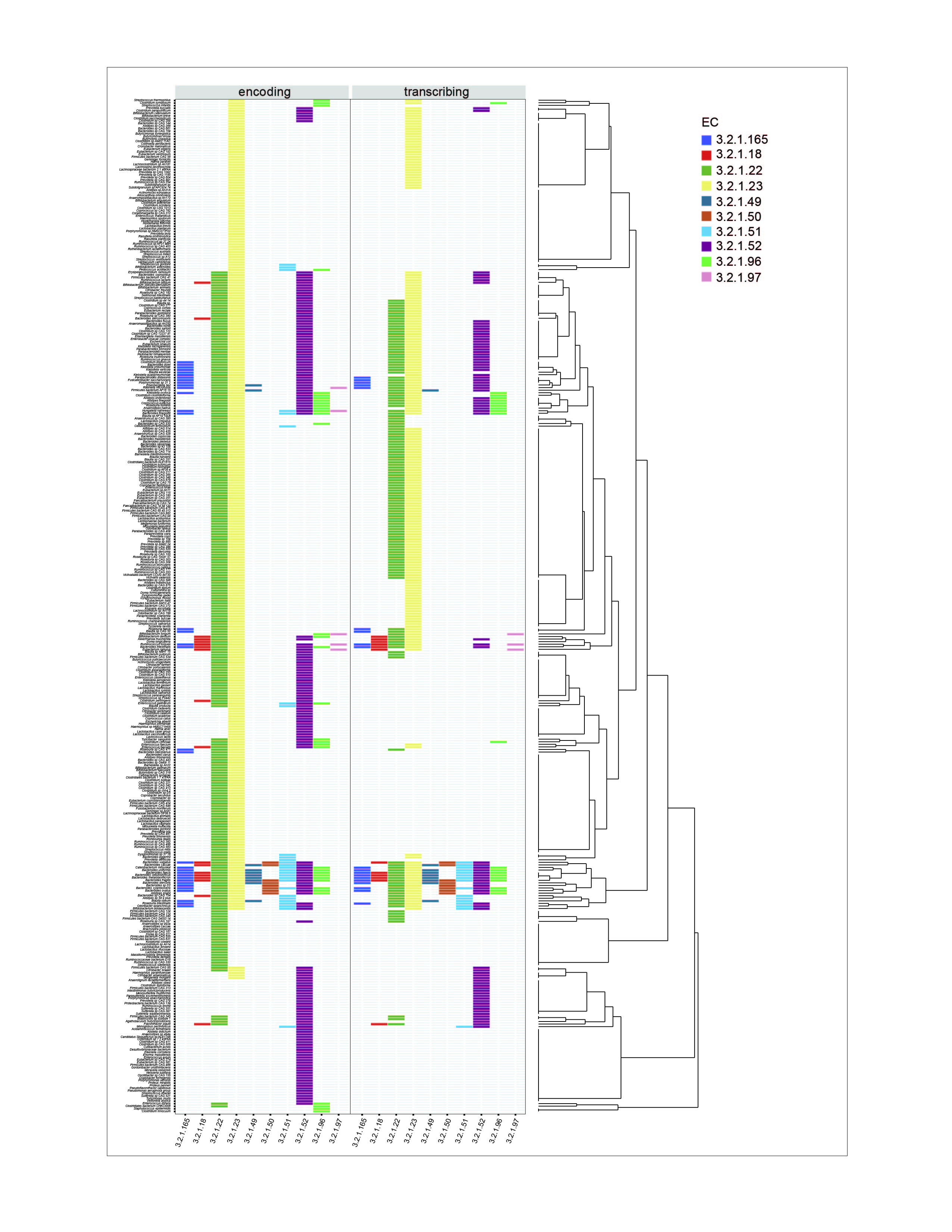

Supplement: fiaf118_Supplemental_Files [file fiaf118_supplemental_files.zip › Supplementary_Figure_1.jpg]

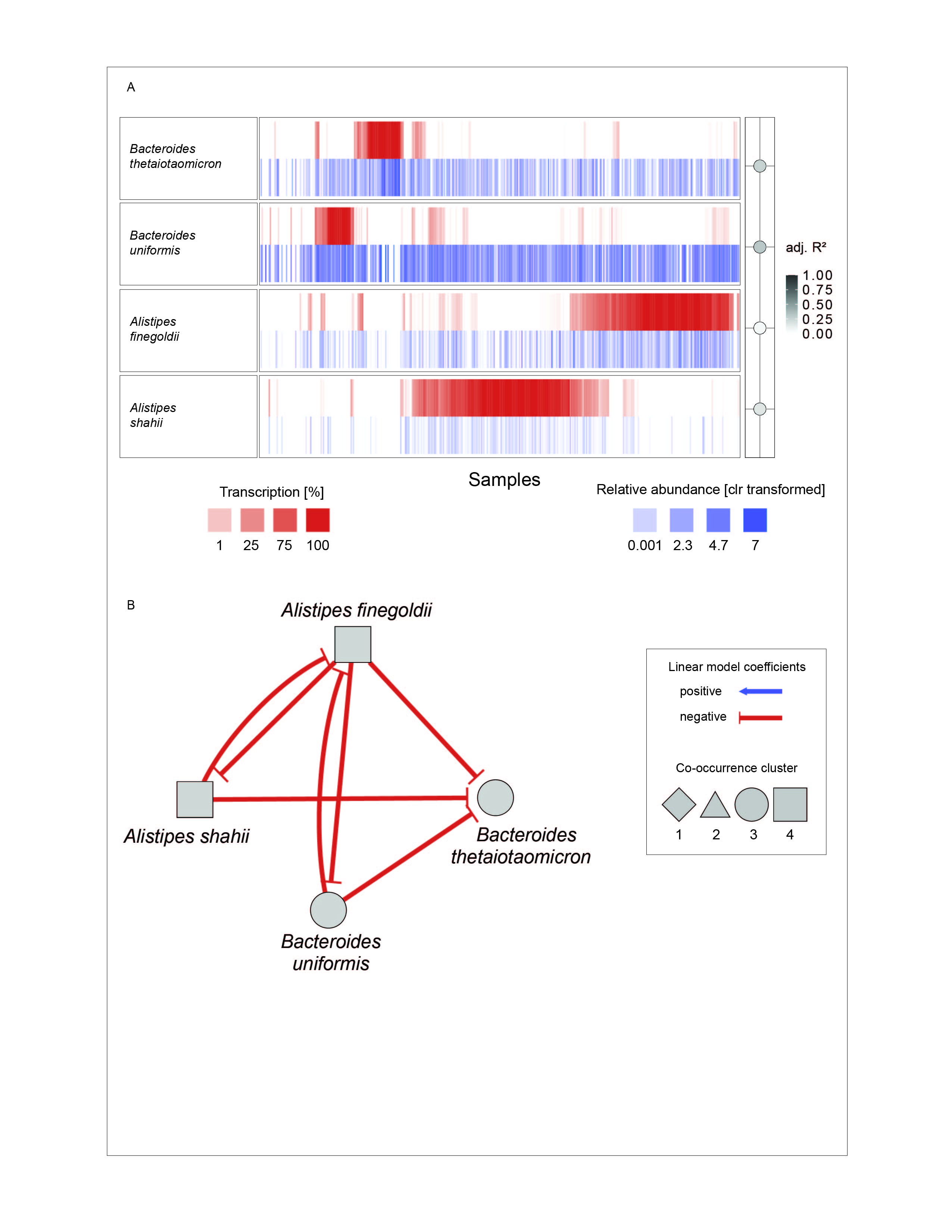

Supplement: fiaf118_Supplemental_Files [file fiaf118_supplemental_files.zip › Supplementary_Figure_10.jpg]

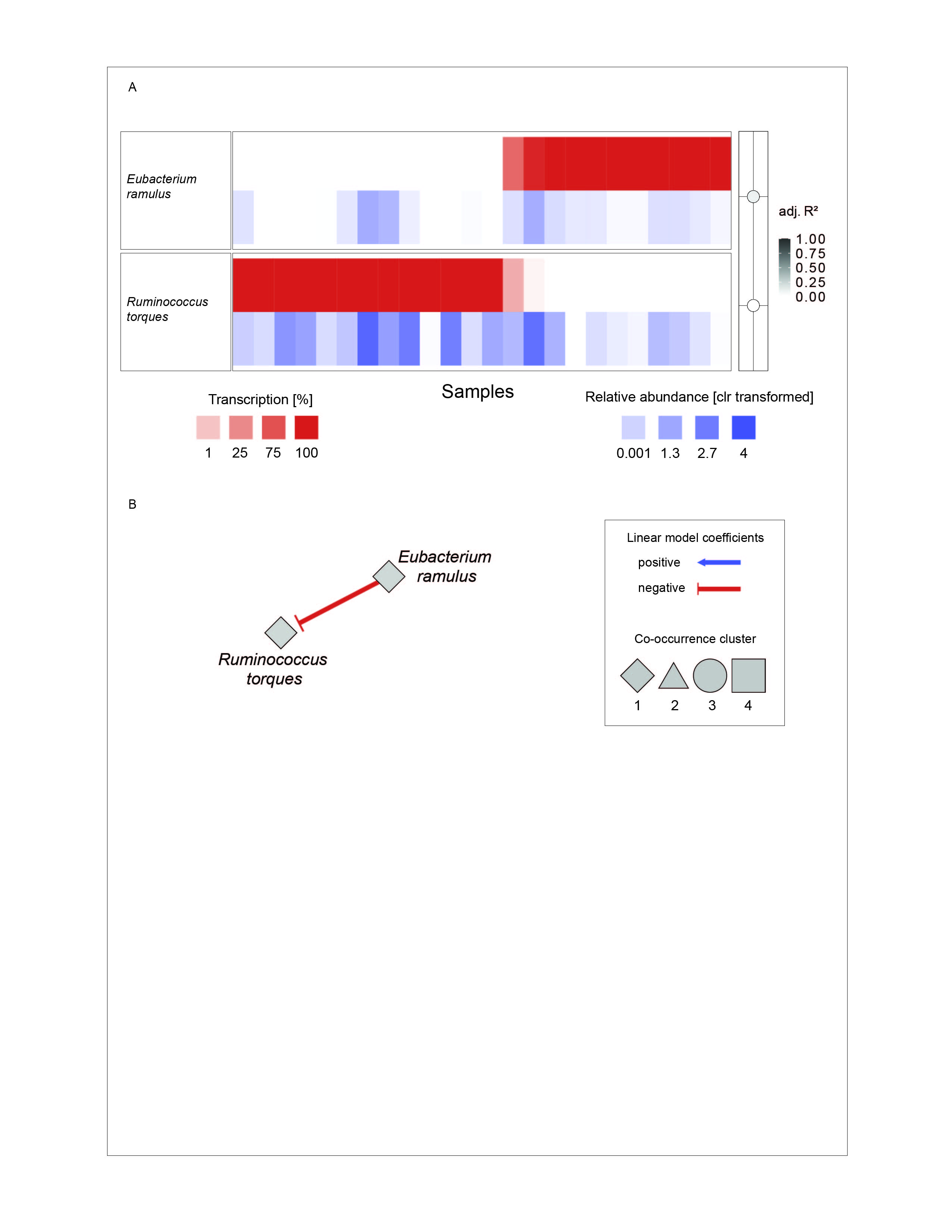

Supplement: fiaf118_Supplemental_Files [file fiaf118_supplemental_files.zip › Supplementary_Figure_11.jpg]

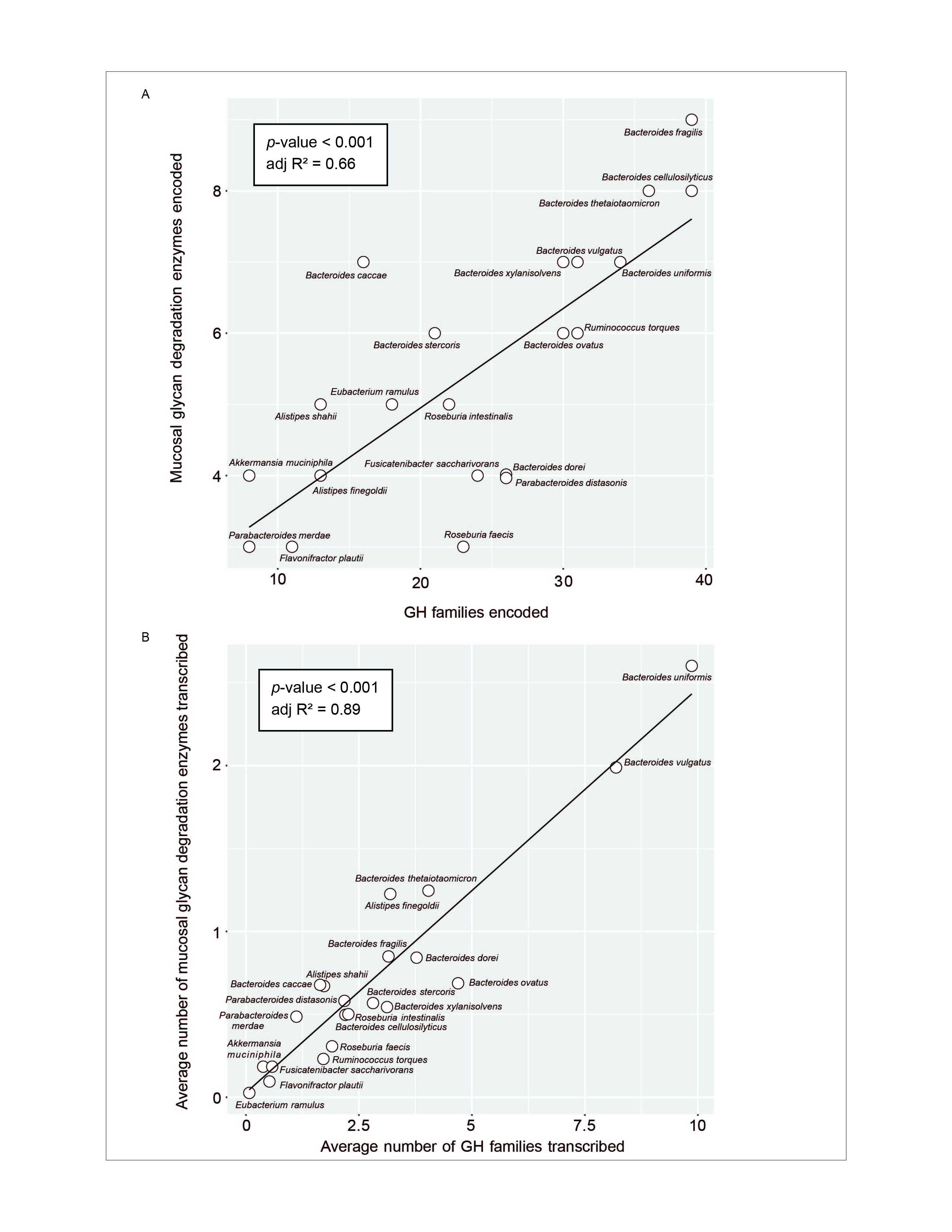

Supplement: fiaf118_Supplemental_Files [file fiaf118_supplemental_files.zip › Supplementary_Figure_12.jpg]

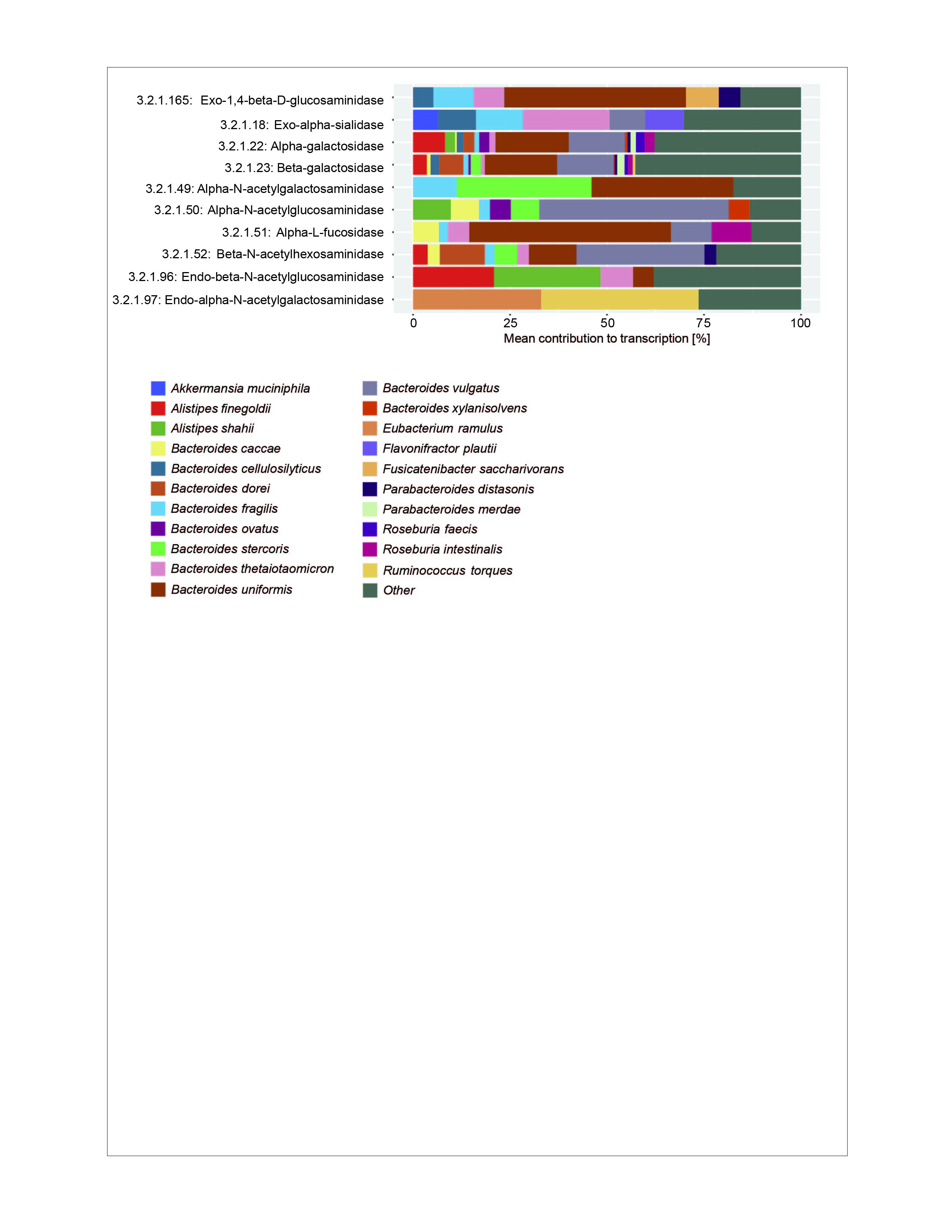

Supplement: fiaf118_Supplemental_Files [file fiaf118_supplemental_files.zip › Supplementary_Figure_2.jpg]

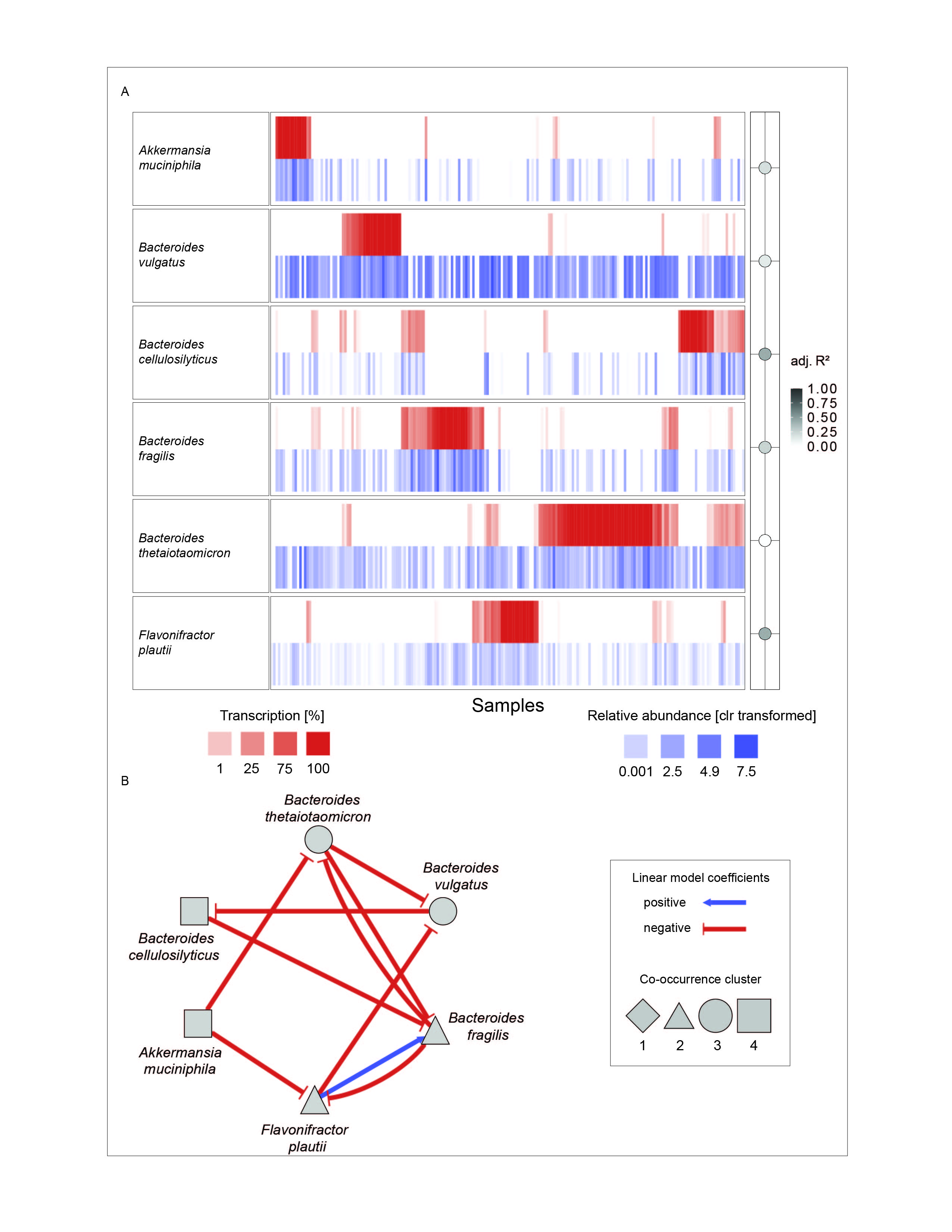

Supplement: fiaf118_Supplemental_Files [file fiaf118_supplemental_files.zip › Supplementary_Figure_3.jpg]

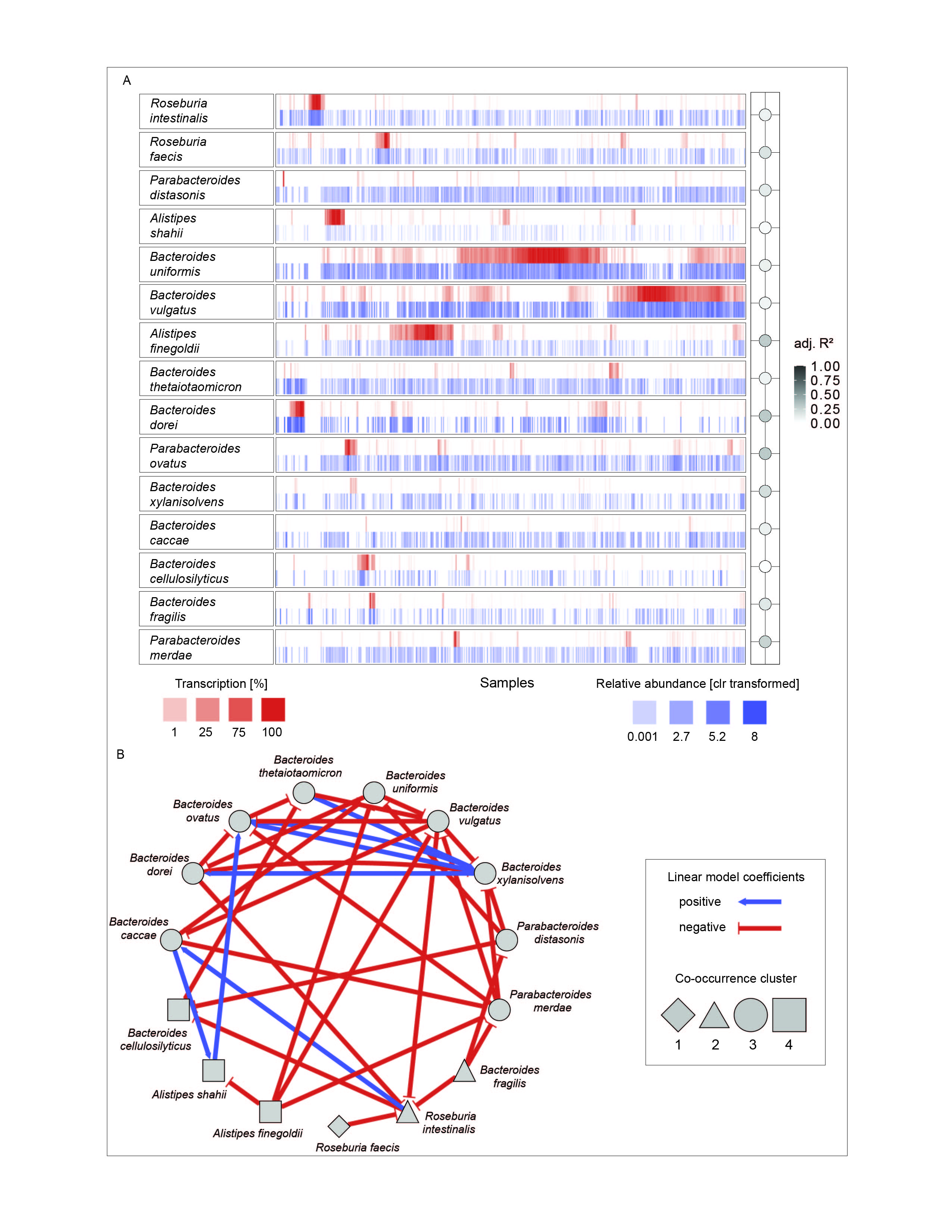

Supplement: fiaf118_Supplemental_Files [file fiaf118_supplemental_files.zip › Supplementary_Figure_4.jpg]

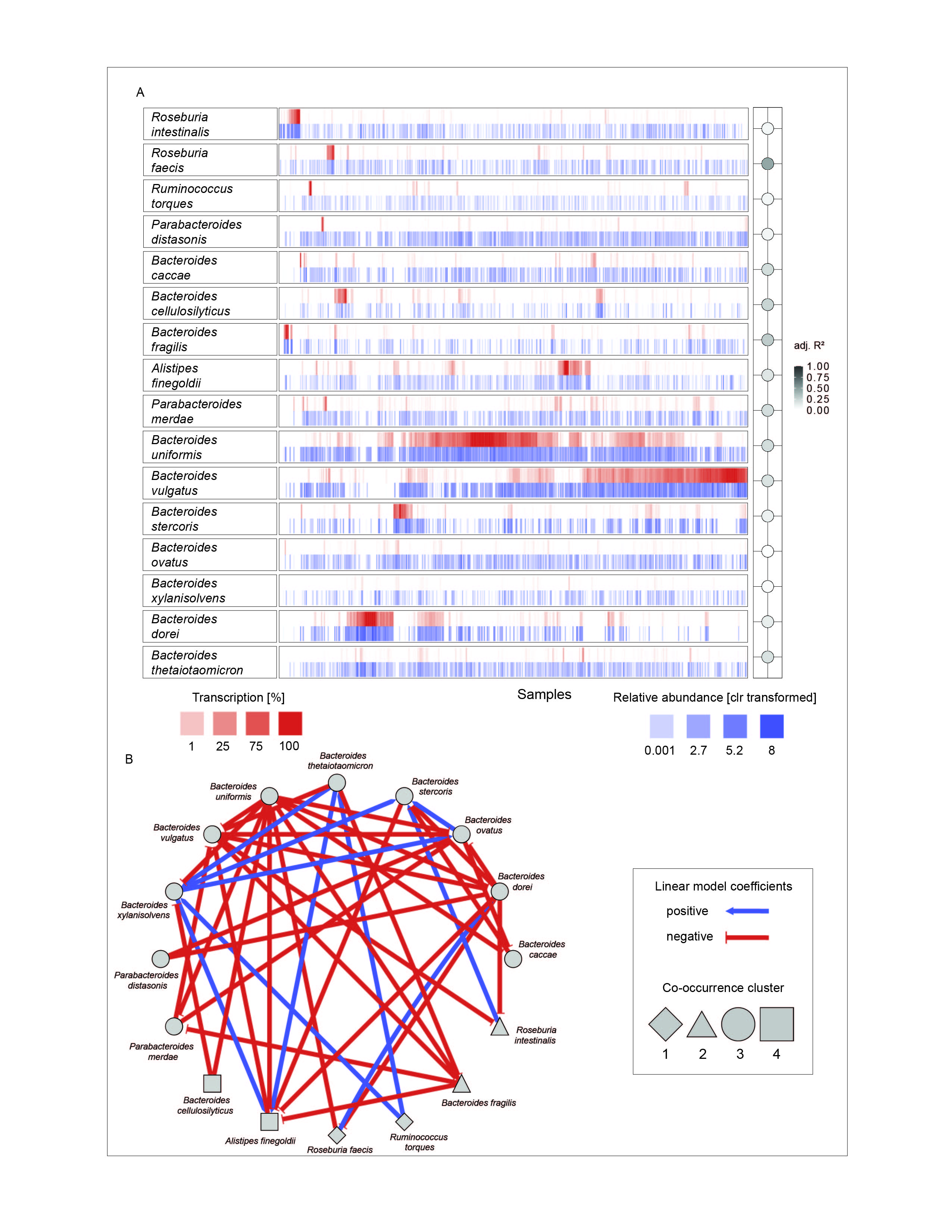

Supplement: fiaf118_Supplemental_Files [file fiaf118_supplemental_files.zip › Supplementary_Figure_5.jpg]

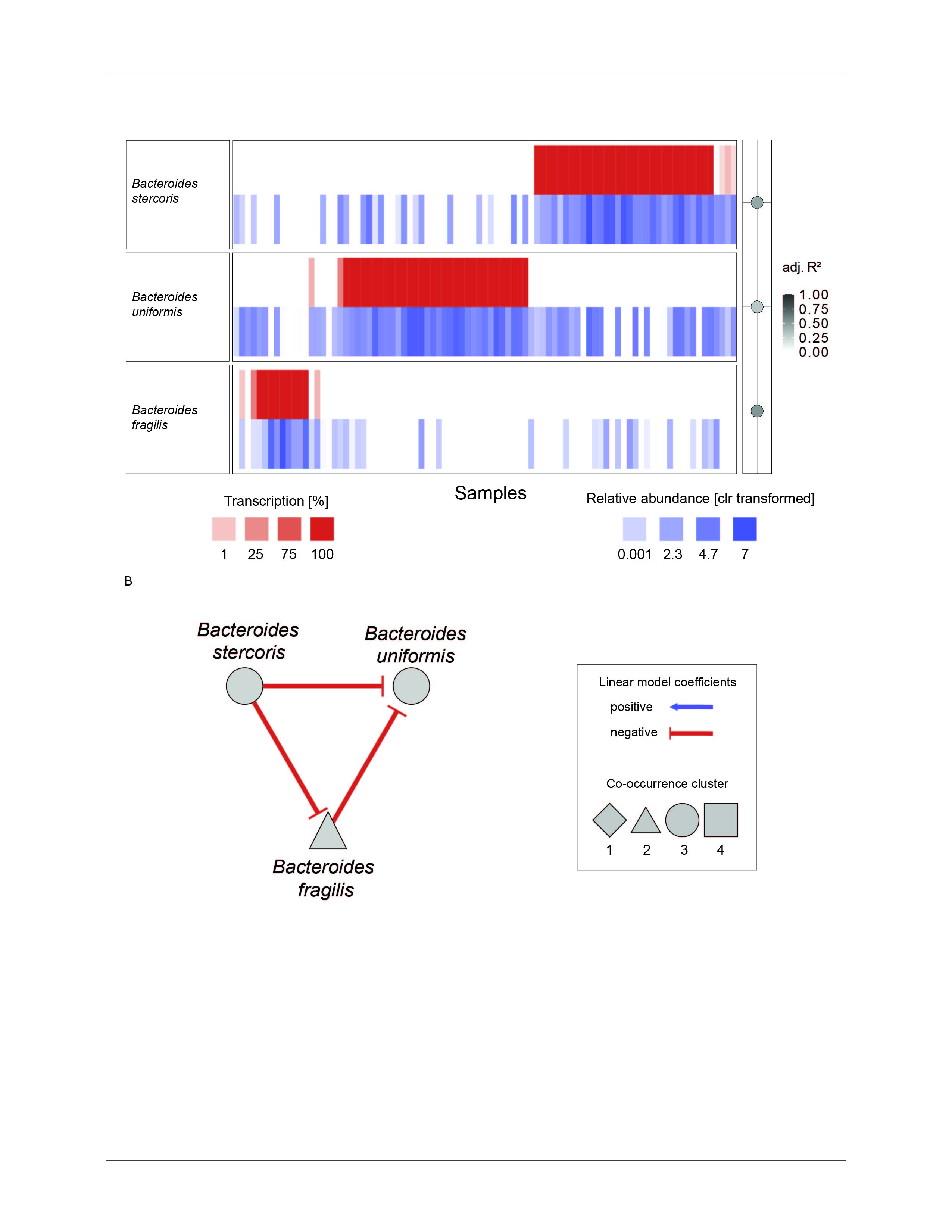

Supplement: fiaf118_Supplemental_Files [file fiaf118_supplemental_files.zip › Supplementary_Figure_6.jpg]

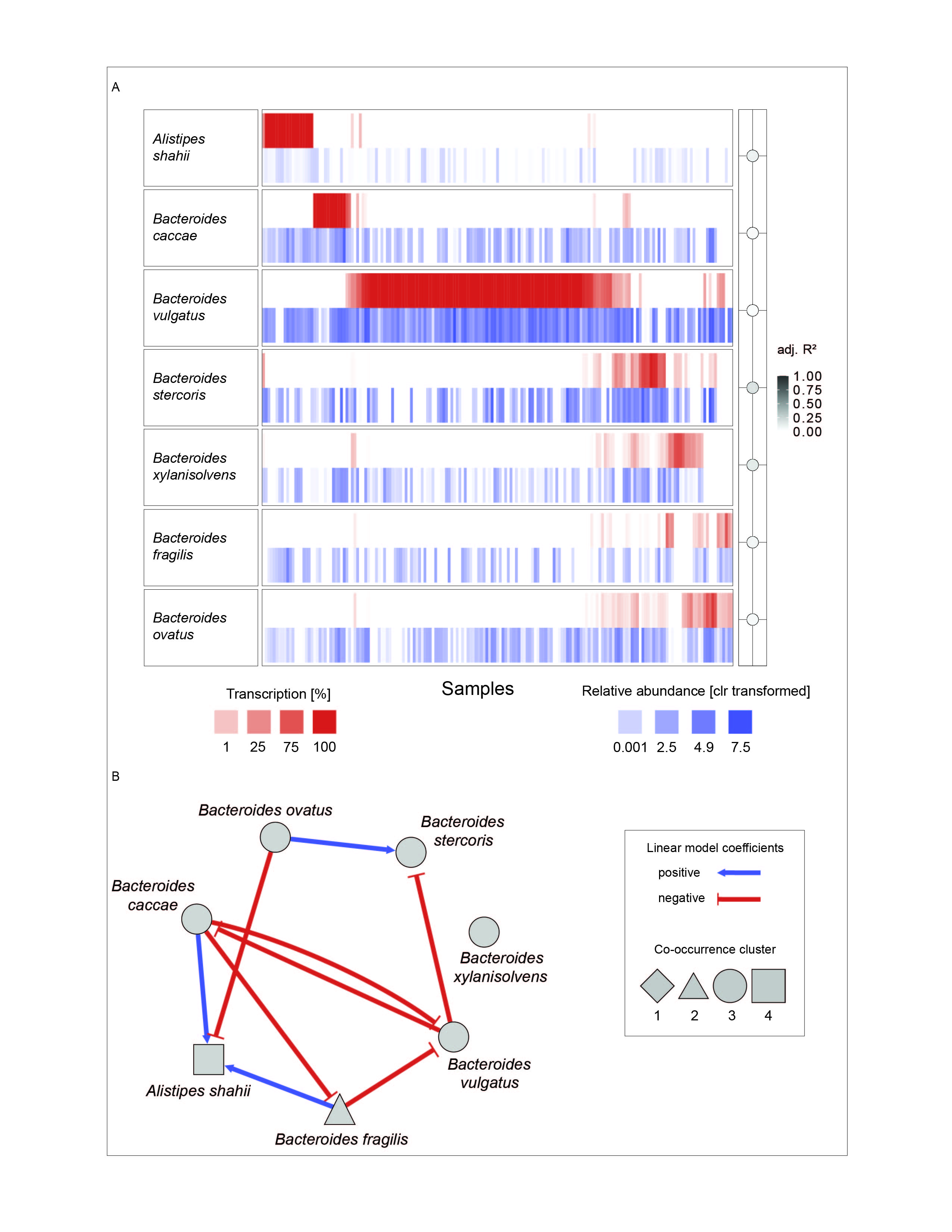

Supplement: fiaf118_Supplemental_Files [file fiaf118_supplemental_files.zip › Supplementary_Figure_7.jpg]

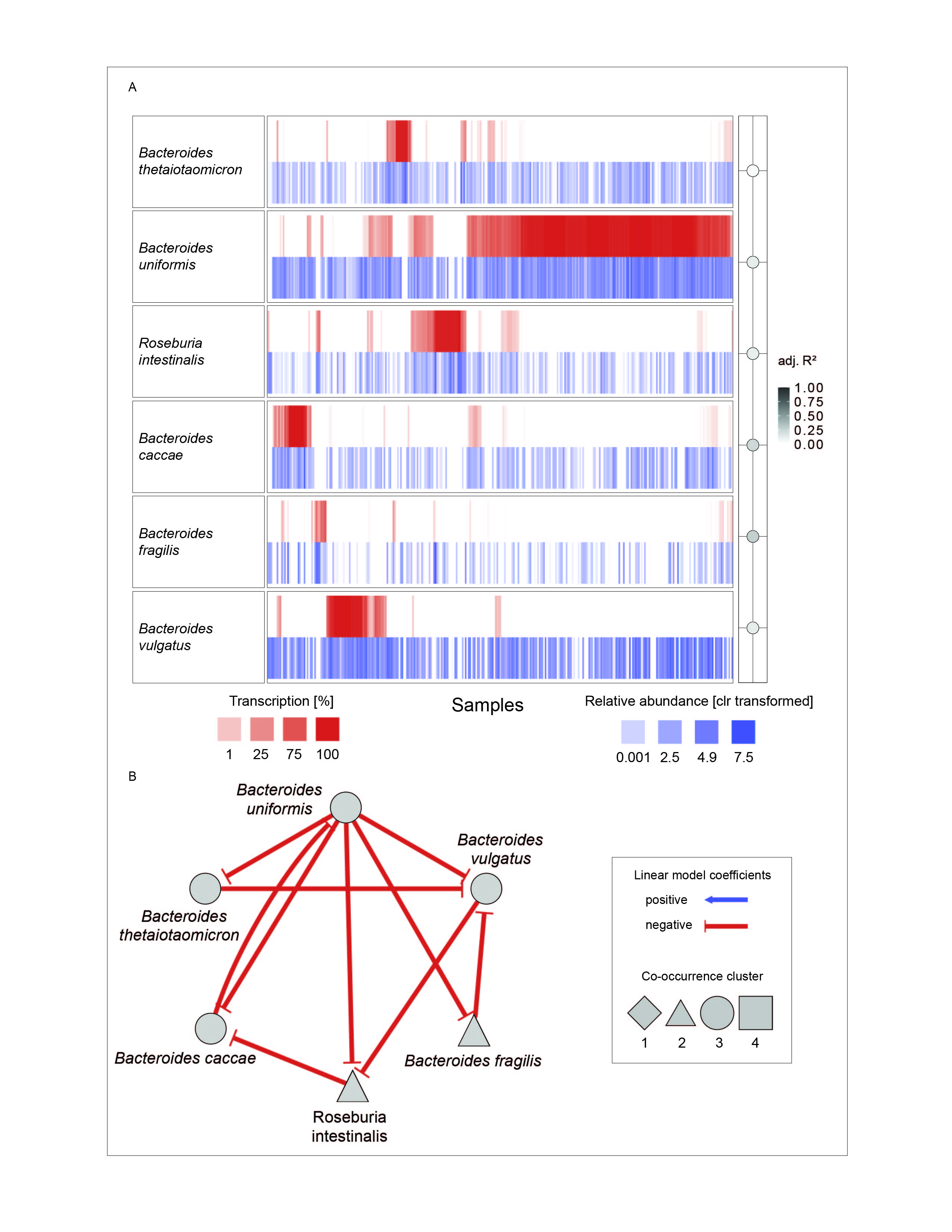

Supplement: fiaf118_Supplemental_Files [file fiaf118_supplemental_files.zip › Supplementary_Figure_8.jpg]

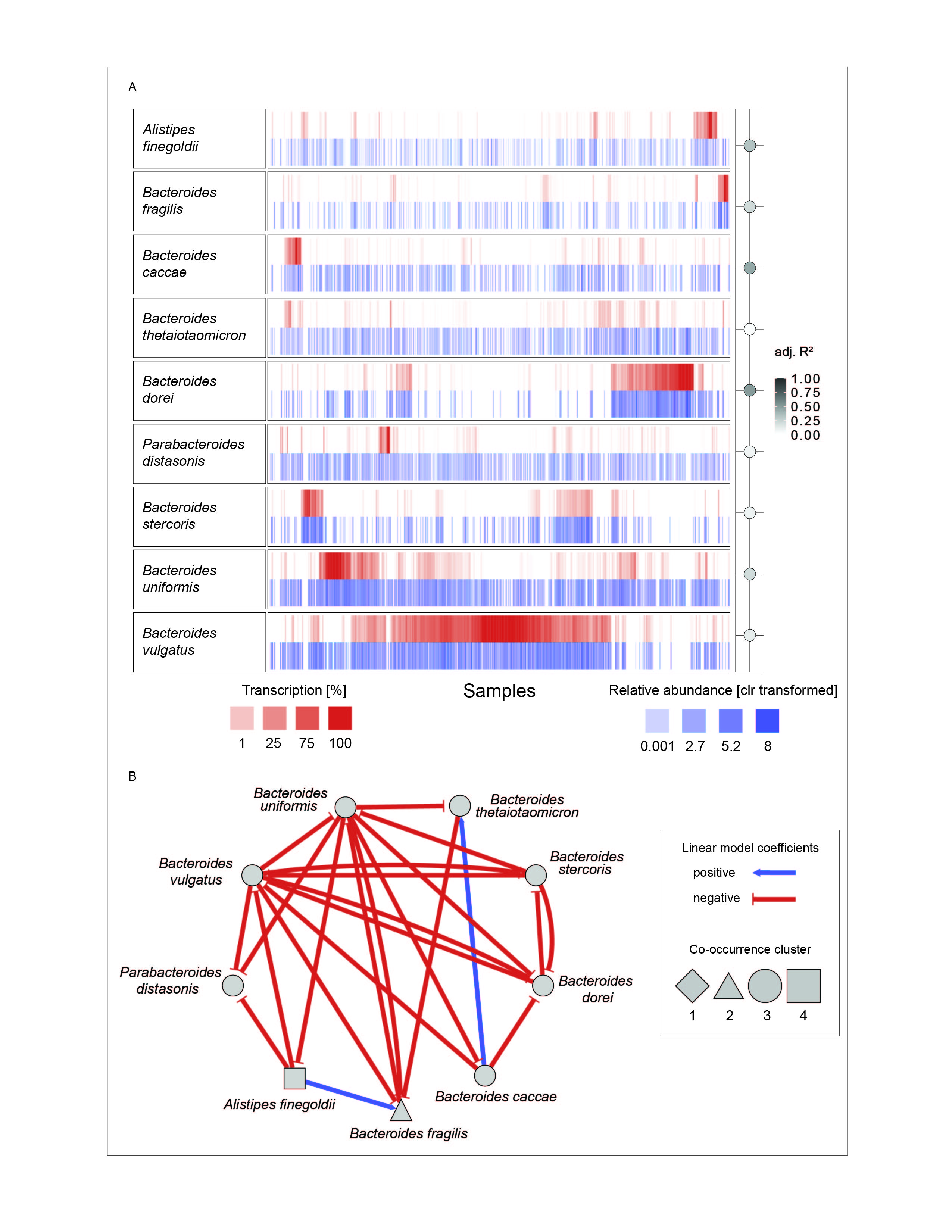

Supplement: fiaf118_Supplemental_Files [file fiaf118_supplemental_files.zip › Supplementary_Figure_9.jpg]
